# System Prediction and Validation of TCM for Chronic Myeloid Leukemia Treatment from the Perspective of Low-Toxicity Chemotherapy: A Stilbene *α*-Viniferin Has a Proapoptotic Effect on K562 Cells via the Mitochondrial Pathway

**DOI:** 10.1155/2020/1986962

**Published:** 2020-02-10

**Authors:** Bing-yang Chai, Fu-Kai Gong, Ze-Hui Chen, Zhao-Xue Li, Bo Zhang

**Affiliations:** ^1^Key Laboratory of Xinjiang Endemic Phytomedicine Resources, Ministry of Education, Shihezi University, Shihezi 832002, Xinjiang, China; ^2^School of Pharmaceutics, Shihezi University, Shihezi 832002, Xinjiang, China

## Abstract

**Objective:**

In traditional Chinese medicine (TCM), chronic myeloid leukemia (CML) has been attributed to “poisoned bone marrow,” which is viewed as a loss of Qi or blood, a deficiency in Yin or Yang that causes a complex imbalance between cell growth and death. Malignant myeloid progenitor cells display excessive growth that is difficult to control without toxicity. More than 60 herbs in TCM have shown efficacy against CML. However, the key molecules and mechanisms involved in the holistic-level characterization, as well as the effective target associations, are still unknown.

**Methods:**

The present study employed a computational approach with filtering potential compounds via admetSAR, systems biology-based functional data prediction, and biochemical and molecular biological validation.

**Results:**

We generated 118 bioactive compounds from 11 herbs within four dialectical therapy groups that are most commonly used to treat CML and predicted 141 potential targets. The stilbene resveratrol and its derivatives were found to be highly related to these targets. Among them, *α*-viniferin was predicted to target Bcl-2, caspase-3, 8, and 9, MAPK14, CDK2, HSP90AA1, and others, reflecting CML therapeutic strategies. *In vitro*, experimental data showed a nonnecrotic growth limitation of K562 cells caused by *α*-viniferin was predicted to target Bcl-2, caspase-3, 8, and 9, MAPK14, CDK2, HSP90AA1, and others, reflecting CML therapeutic strategies. *μ*g·mL^−1^ at 24 h. Finally, we validated the chemotherapeutic effect of *α*-viniferin was predicted to target Bcl-2, caspase-3, 8, and 9, MAPK14, CDK2, HSP90AA1, and others, reflecting CML therapeutic strategies.

**Conclusions:**

Our work sheds light on the mechanism of the efficacy of the stilbene *α*-viniferin in TCM for the prevention of CML. This work also predicts and validates targets in the mitochondrial signaling pathway, providing a novel strategy for CML treatment.*α*-viniferin was predicted to target Bcl-2, caspase-3, 8, and 9, MAPK14, CDK2, HSP90AA1, and others, reflecting CML therapeutic strategies.

## 1. Introduction

Chronic myelogenous leukemia (CML) is a hematologic malignancy of stem and progenitor cells that accounts for 15% of adult leukemia cases, with a global annual incidence of 1.6–2.0/10 million [1]. In the Chinese population, CML accounts for 20% of all cases of leukemia and 95% of cases of chronic leukemia, with an annual incidence of 0.36/10 million [2].

Currently, the BCR-ABL gene mutation is considered the main molecular mechanism of CML. The translocation (9;22) q (34;11) entails the movement of the Abl proto-oncogene to the break-point cluster on the chromosome 22 long arm area (BCR) to form the BCR-ABL fusion gene [3]. The fusion gene encodes a protein with tyrosine kinase activity, leading to the expansion of the pool of myeloid progenitor cells. Myeloid progenitor cells display excessive growth as a hematologic disorder, which is difficult to treat without toxicity. Chemotherapy is the main treatment of CML, and the tyrosine kinase BCR-ABL is the main target. BCR-ABL has become the focus of drug research and development, of which significant advances have been made. Drugs such as imatinib, nilotinib, and dasatinib inhibit the phosphorylation activity of BCR-ABL and activation of downstream molecules, thus limiting the growth of CML cells [4]. However, the high costs make many medium- and low-income people unable to afford it. During chemotherapy, the targets of these drugs are associated with serious gastrointestinal toxicity, severe diarrhea, and blood toxicity.

In order to find new CML treatments, natural products in traditional Chinese medicine (TCM) have become attractive clinical candidates because of their long-proven clinical use with therapeutic effects in China [5]. In TCM theory, CML is viewed as an imbalance of many integral body processes that coordinate with each other, complement each other functionally, and influence each other pathologically. In long-term TCM clinical practice, a unique theory has been established for the treatment of hematological diseases, which is focused on the rebalance of the Qi and blood and of Yin and Yang. Thus, CML is believed to result from “poisoned bone marrow,” which is observed as a loss of Qi or blood or a deficiency in Yin or Yang, resulting in a complex imbalance [6]. According to the principles of disease and syndrome classification, the general treatments and prescriptions for CML are divided into the following four dialectical therapy groups: “clearing heat and cooling blood,” “relieving poisoning and detoxification,” “eliminating wind and dispersing wet,” and “supplementing Qi and nourishing Yin” [7].

The long history and multiple target effects of Chinese herbal medicine make the efficacy of TCM more durable and milder, with fewer by-effects. The use of Chinese medicine in the treatment of leukemia began as early as the 1960s. *Paeonia anomala L.*, *Rheum palmatum L.*, *indigo*, and others have been found to have efficacy for the treatment of leukemia [8–10]. The clinical application of TCM has been accepted for CML treatment in western China because of its fewer associated adverse reactions and low cost. It is widely used in local hospitals and has achieved good clinical outcomes [11, 12]. TCM with active *indigo* ingredients has been successfully applied as an antileukemia drug. Although nearly hundreds of prescriptions for CML treatment have been recorded, the mechanisms of most TCM drugs remain unclear.

Recently, network pharmacology, genomics, proteomics, and metabolomics provide holistic approaches to study the essence of TCM and the functions of herbal compounds [13]. Meanwhile, systems biology-based analysis of TCM and drug-target interaction network approaches has made a significant contribution to study TCM [14]. This approach provides a possibility for a multitarget model study of TCM treatment for leukemia. Systems pharmacology studies of some TCM formulas have shown good results, such as that for the application of the Chinese herbal *Radix Curcumae* to treat cardiovascular disease, and these studies have provided insights into cardiovascular drug discovery and therapy [15]. The key molecules and mechanisms underlying CML treatment must be elucidated on a holistic level. Thus, we aimed at characterizing the active ingredients and target associations via both systems pharmacological and molecular biological methods, using an *in silico* approach combined with an *in vitro* approach to uncover the mechanism of TCM therapy in CML.

## 2. Materials and Methods

### 2.1. Materials

Hoechst 33258 (cat no. B8030) and RIPA lysate (cat no. R0010) were purchased from Solarbio Science and Technology Co., Ltd. (Beijing, China). Acridine orange (AO), dimethyl sulfoxide (DMSO), and ethidium bromide (EB) were purchased from Sigma-Aldrich (St. Louis, MO, USA). RPMI 1640 medium was purchased from Gibco BRL (Grand Island, NY, USA; cat no. 31800-022). Fetal bovine serum (FBS) was purchased from Biological Industries (Israel; cat no. 1413865). The primary antibody of Bcl-2, Bax, and *β*-actin, and its corresponding secondary antibody were purchased from Boster (China). FITC Annexin V and PI were purchased from BD Biosciences (Franklin Lakes, NJ, USA; cat no. 556547).

### 2.2. Chemical Library Construction

All herbal chemicals used in the treatment of CML were collected from (1) the TCMSP database (http://lsp.nwu.edu.cn/tcmsp.php), (2) the Chinese Academy of Sciences' Chemistry Database (http://www.organchem.csdb.cn), (3) the Chinese Herbal Drug Database, and (4) the literature. And the canonical SMILES format of all herbal chemicals was collected from the NCBI PubChem database (http://www.ncbi.nlm.nih.gov/pccompound).

### 2.3. *In Silico* Screening for Potential Active Molecules via admetSAR Prediction

To find the potential active molecules in CML-related TCM, all collected chemicals in the library were screened according to the predicted *in silico* oral bioavailability records via admetSAR (http://lmmd.ecust.edu.cn/admetsar1) [16]. The compounds with negative parameters of human intestinal absorption (HIA) and Caco-2 permeability (Caco-2) and negative metabolism parameters of inhibiting the five major CYP isoforms (CYP450 1A2, CYP450 2C9, CYP450 2C19, CYP450 2D6, and CYP450 3A4) were discarded, thereby remarkably reducing a great quantity of primitive chemicals to a smaller group, which was used to develop a TCM formula [17]. The inhibitory parameters of the five major CYP isoforms which contribute to the metabolism of drugs were calibrated comprehensively and calculated with the following equation [18]:

(1) $$\begin{array}{c} \text{score}=\Sigma k\left(\text{result}\right)\left(Q\right). \end{array}$$

Equation (1) adds together the results and overall predictive accuracy (*Q*) of each compound, with five major CYP isoforms (index *k*). The overall predictive accuracies (*Q*) of five major CYP isoforms (CYP450 1A2, CYP450 2C9, CYP450 2C19, CYP450 2D6, and CYP450 3A4) were 0.8147, 0.8018,  0.8551, 0.8054, and 0.6450, respectively [19].

### 2.4. Activities of Target Molecules and Network Construction

To identify CML-specific target, we collected CML targets from TCMSP, DisGeNET (http://210.107.182.61/geneSearch/), and the Online Mendelian Inheritance in Man (OMIM) database [20]. To elucidate the effects of drugs, we collect compound targets to study compound-target interactions. The corresponding targets are obtained from TCMSP database searching by compound name and PubChem database on Document Mining. In addition, the predicted targets are further screened using the sysDT drug target prediction model [21]. To explain the relationship between candidate compounds and predicted targets and disease, we established “candidate compound-candidate target network (cC-cT network),” and “target-disease network” (T-D network), and all target networks were created by Cytoscape (http://www.cytoscape.org/). In the network, compounds and targets are represented by nodes, and interactions between two nodes are represented by edges [22]. The degree of a node is the number of edges connected to the node. The greater the degree is, the more nodes in the network that are directly related to the node, indicating that the node is more important in the network.

### 2.5. Cell Culture

The human CML K562 cell line was obtained from Cancer Cell Repository (Shanghai Cell Bank, Shanghai, China). K562 cells were cultured in RPMI-1640 containing 10% fetal bovine serum and 100 U·mL^−1^ each of penicillin and streptomycin in a humidified atmosphere maintained at 37°C and 5% CO_2_.

### 2.6. Analysis of Cell Viability

The cells were inoculated with 4 × 10^4^/mL per well in a 96-well culture plate, and the volume of the culture medium was 200 *μ*L. Different concentrations of *α*-viniferin were added and cells were cultured at 37°C for 12, 24, and 48 h. 20 *μ*L of MTT solution (5 g/L) was added to each well, the cells were continued to culture for 4 h and centrifuged at 1000 r/min for 10 min, and the supernatant was discarded and 150 *μ*L of DMSO was added. After shaking for 10 min, a plate reader (Varioskan Flash 3001, USA) detects the A value of each well at 490 nm and calculates the cell proliferation inhibition rate according to the following formula:

(2) $$\begin{array}{c} \text{inhibition rate }\left(\text{\%}\right)=\frac{\left(\text{control group A value}-\text{experimental group A value}\right)}{\text{control group A value}}\times 100\text{\%}. \end{array}$$

### 2.7. Sub-Intracellular Structure Observation

The cells were inoculated with a density of 5 × 10^5^ cells/well in 6-well plates and treated with or without *α*-viniferin for 24 h at 37°C. Other steps are as follows: (1) fixation: cells were fixed by glutaraldehyde 2.5% at 4°C for several weeks and they were rinsed gently with 0.1 mol·L^−1^ phosphate-buffered saline (PBS). Then, cells were fixed with 1% osmium tetroxide for 3 h and they were rinsed gently with PBS; (2) dehydration: cells were dehydrated by serial dilution of ethanol (50, 70, 90, and 100%, 30 minutes each time, and 100% three times); (3) embedding: cells were embedded in epoxy resin; (4) curing and slice; (5) 3% uranyl acetate-lead lead citrate double staining; and (6) transmission electron microscope (JEM-1230; Jeol, Tokyo, Japan) observation.

### 2.8. Analysis of Cell Apoptosis

To detect morphological changes in nuclear chromatin of apoptotic cells using Hoechst 33258 and AO/EB staining, K562 cells treated with drugs or untreated were collected and washed with PBS. Then, the cells were stained with either 10 mg·mL^−1^ Hoechst 33258 or 100 mg·mL^−1^ AO/EB double staining in PBS. Fluorescence microscopy (Axio Observer A1; Zeiss, Germany) was used to record apoptotic and necrotic cells.

To analyze apoptosis, K562 cells were stained with FITC Annexin V and PI and a flow cytometer was used (Calibur; BD Biosciences, Franklin Lakes, NJ, USA) to measure fluorescence.

### 2.9. Determination of Mitochondrial Membrane Potentials

For JC-1 analysis, the K562 cells were treated with or without different *α*-viniferin concentrations for 24 h. We analyzed mitochondrial membrane potentials by JC-1. We analyzed samples by measuring the fluorescence value of flow cytometry (FACS Calibur; BD Biosciences).

### 2.10. Evaluation of Bcl-2 Family Gene Expression

To analyze the gene expression levels of the Bcl-2 family, the total RNA was extracted from cells using a commercial kit (Sangong Co., Shanghai, China). RNA quality was assessed using the A260/A280 ratio and 1.5% agarose gel electrophoresis. RNA was converted to cDNA using a PrimeScript 1^st^ strand cDNA Synthesis Kit according to the manufacturer's instructions (Takara Bio Inc., Japan). The PCR primers [23, 24] were synthesized by Sangong Co. Ltd. (Shanghai, China), and the forward and reverse primer sequences were as follows: 5′-GTTCCAGATCCCAGAGTTTG-3′ and 5′-CCTCCATGATGGCTGCTG-3′ for Bad, 5′-TTTCTCACGGCAACTTCAAC-3′ and 5′-GGAGGAAGTCCAATGTCCAG-3′ for Bax, 5′-GAGGATTGTGGCGTTCTTT-3′ and 5′-CCCAGCCTCCGTTATCCT-3′ for Bcl-2, 5′- ACATCCCAGCTCCACATCAC-3′ and 5′-CGATCCGACTCACCAATACC-3′ for Bcl-xL, and 5′- TCAACGACCACTTTGTCAAGCTCA-3′ and 5′-GCTGGTGGTCCAGGGGTCTTACT-3′ for GAPDH.

Detection of Bcl-2 family gene expression was carried out using quantitative real-time RT-PCR (qRT-PCR), which was performed using a single-tube SYBR Green Kit (QIAGEN, Valencia, CA, USA), a Rotor-Gene Q Real-Time PCR System (Rotor-Gene Q, QIAGEN), and specific primer sets. Bcl-2 family mRNA expression was calculated by the 2^−△△Ct^ method, and glyceraldehyde-3-phosphate dehydrogenase (GAPDH) was used as an endogenous control.

### 2.11. Evaluation of Bcl-2 Family Protein Expression

K562 cells were treated with or without different *α*-viniferin concentrations for 24 h, and then RIPA lysate was used to extract protein. The levels of Bcl-2 and Bax were analyzed using western blotting with the primary antibody (1 : 400) and its corresponding secondary antibody (1 : 10000) according to the manufacturer's instructions [25].

### 2.12. Statistical Analyses

All data were used as the mean ± SD of at least three independent experiments and assessed by ANOVA. Student's *t*-test for multiple comparisons was used to identify differences between groups. *P* < 0.05 was considered to be statistically significant value.

## 3. Results

### 3.1. Compounds Collecting and Sorting in CML Herbs

In the Chinese ethnopharmacological system, CML treatments can be divided into four dialectical therapy groups according to syndrome differentiation, and they are highly connected to imbalances in the following four elements: Qi, blood, Yin, and Yang [26]. According to the four dialectical therapy groups, we collected prescriptions for treating CML (Supplemental [Supplementary-material supplementary-material-1]), from the database of traditional Chinese medicine prescriptions (https://db.yaozh.com/fangji) and Chinese prescription database in National Scientific Data Sharing Platform for population and health (http://cowork.cintcm.com/engine/outline?page=2&channelid=37595&ispage=yes). The databases contain the information of prescriptions contained in Treatise on Typhoid Fever, Synopsis of the Golden Chamber, Criteria of Syndrome and Treatment, Orthodox Surgery, and other books. The 11 most frequently used herbs in CML treatments are classified as follows: clearing heat and cooling blood (“*Rheum palmatum L.*, *Reynoutria japonica Houtt.*, *Moutan officinalis (L.) Lindl. & Paxton, cortex*, *Paeonia anomala L.*”); relieving poisoning and detoxifying (“*Smilax China L.*, *Smilax glabra Roxb.*”); eliminating wind and dispersing wet (“*Notopterygium incisum* K.C.Ting ex H.T.Chang, *Morus alba L., twig, dry* and *Eucommia ulmoides Oliv.*”); and nourishing Yin and invigorating Yang (“*Stemona sessilifolia (Miq.) Miq.* and *Ginkgo biloba L., leaf, dry*”). Recently, there has been increasing attention on validating and explaining the combined principles of TCM by modern approach. In those studies, a systems pharmacology method has been introduced and applied to investigate TCM, which may allow for the understanding of the combined effects of herbal formulas from the ADMET *in silico*, active chemical, target protein, and disease network perspectives [5]. We predicted the absorption and metabolism-associated properties of all compounds via admetSAR.

The absorption showed positive results for 121 of 981 chemicals (12.33%) based on HIA and Caco-2 models. Of these herbs, *Ginkgo biloba L.* (23 compounds), *Notopterygium incisum* K.C.Ting ex H.T.Chang (22 compounds), and *Rheum palmatum L.* (17 compounds) possessed the most compounds with good absorption (Supplemental [Supplementary-material supplementary-material-1]). Thirty-two candidate compounds also met the positive metabolic properties and pharmacokinetic data (Table 1).

### 3.2. Candidate Compounds-Candidate Targets (cC-cT) Networks

**Figure 1:**
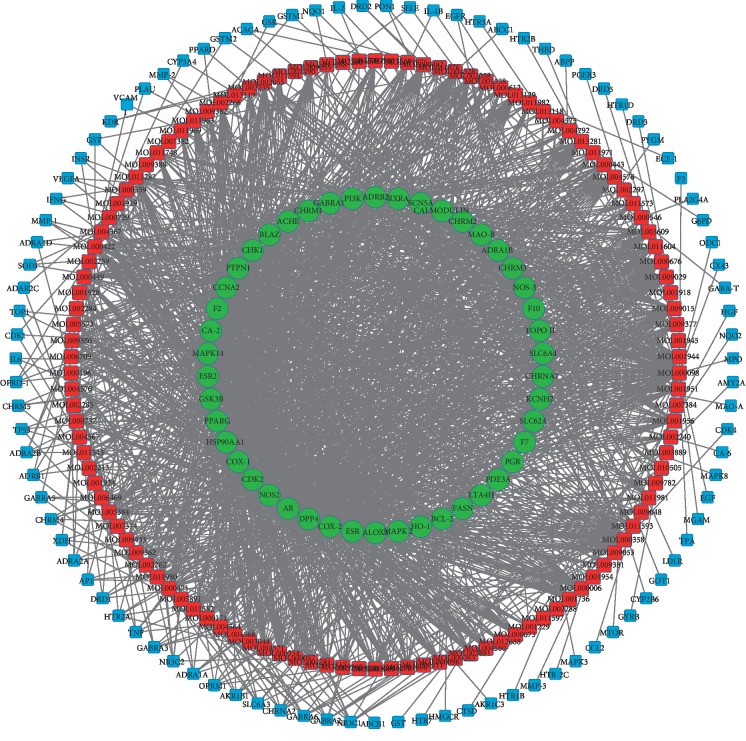
Network of 118 candidate compounds and 141 candidate targets. The red circles represent the compounds, whereas the green and blue circles delineate the targets.

Systems pharmacology can also provide new approaches for drug discovery for complex diseases. By considering drug actions in the context of the regulatory networks within which the drug targets and disease gene products function, network analysis has the potential to greatly increase our knowledge of the mechanisms underlying the multiple actions of drugs [27]. Here, we used a systems pharmacology method to determine the active components of TCM in the treatment of CML. The cC-cT network has 259 nodes including 118 candidate compounds and 141 candidate targets (Figure 1). The targets in the outer circle exhibited considerably fewer interactions with the candidate compounds than those in the inner circles (the green circles indicate a high degree, whereas the blue circles indicate a low degree). In this result, there were many candidate targets connected to one or two candidate compounds; however, some candidate targets could be affected by multiple compounds rather than a single compound. In the inner circles, the number of targets (degree ≥ 10) was 46 (32% of the total number of targets). The other targets (degree < 10, 68% of the total number of targets) were likely less related to leukemia. The results indicate that, under the network pharmacology research paradigm, candidate compounds and their candidate targets exhibit a relationship in which multiple compounds are acting on multiple targets [5].

### 3.3. T-D Network Analysis

**Figure 2:**
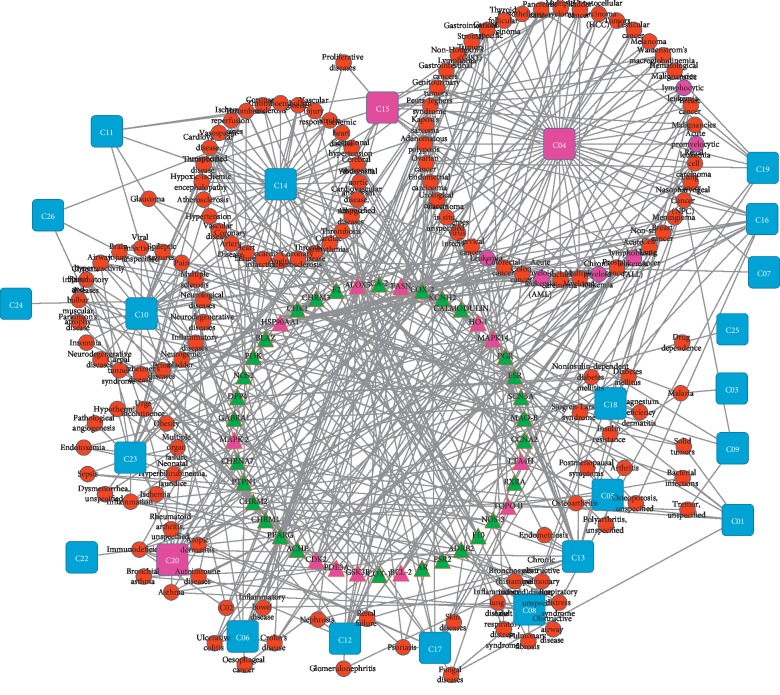
Network of 46 potential targets (triangles) connected to 139 diseases (circles), and diseases are classified into 24 groups (C squares) according to their Medical Subject Headings.

In this section, target-related diseases were searched from GeneCards (http://www.genecards.org) and TTD websites (http://bidd.nus.edu.sg/group/TTD/ttd.asp) and used to construct a T-D network (Figure 2). According to ICD-10 records in Medical Subject Headings (MeSH, http://www.nlm.nih.gov), 139 diseases were classified into 24 groups. For instance, acute myelogenous leukemia and chronic myelogenous leukemia belong to both Neoplasm Diseases (C04) and Hemic and Lymphatic Diseases (C15). To identify the CML disease-associated targets, 1526 targets were collected from TCMSP, DisGeNET, and OMIM. After mapping the disease targets-candidate targets network, we identified 50 targets related to CML (Supplemental [Supplementary-material supplementary-material-1]), and twelve of them belong to 46 potential targets in the inner circles in Figure 1.

We selected 121 chemicals with positive absorption as a prerequisite for analysis. Among them, proapoptotic activities on K562 cells were reported previously. For example, diosmetin induces OCI-AML2 and K562 cell apoptosis via the caspase pathway [28]. Resveratrol induces apoptosis of K562 through p38 and JNK-regulated H2AX phosphorylation [29]. Kaempferol also showed a potent cytotoxic effect on K562 cells and U937 cells through Bcl-2 signaling pathway-induced apoptosis [30].

**Figure 3:**
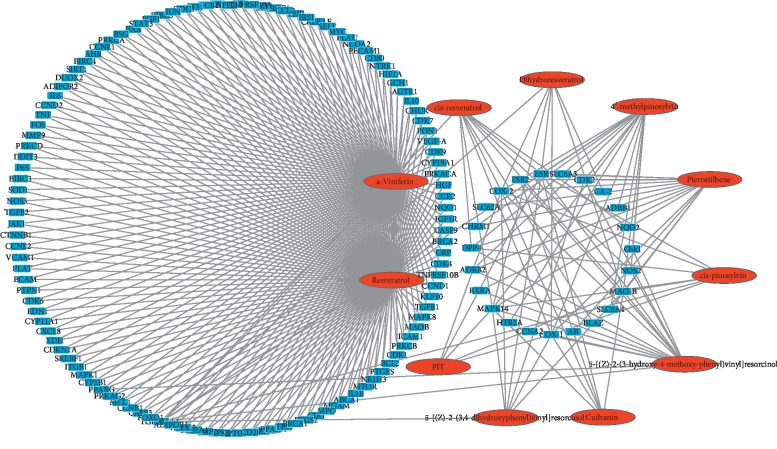
Network of 11 candidate stilbene compounds (cC) and 141 candidate targets (cT). The red circles represent the compounds, whereas the blue circles represent the targets.

Then, we constructed a cC-cT network and found that there were 46 targets with a close relationship to leukemia. From the chemical structure perspective, flavonoid (11 compounds) and stilbene (7 compounds) family were displayed frequently (Supplemental [Supplementary-material supplementary-material-1]). Additionally, literature mining displayed inhibition data of those flavonoids on leukemic cell proliferation, but there have been few reports on stilbenes besides resveratrol. 11 candidate stilbenes from 121 compounds in the whole network of Figure 1 were predicted to have candidate protein targets (Figure 3). We found that *α*-viniferin, a basic resveratrol trimer, was predicted to be closely related to the main targets of leukemia. This finding agrees with the report that resveratrol has antileukemic activities *in vitro* [31]. Next, experiments were performed to clarify the potential antileukemic activity of *α*-viniferin.

### 3.4. cC-cT Networks for Stilbene Compounds

This cC-cT network included 11 stilbenes and their candidate targets, which consists of 152 nodes (11 candidate compounds and 141 candidate targets). The targets in the left blue circle showed interactions with resveratrol (MOL012744) and *α*-viniferin (MOL1808), and those in the right blue circles represent interactions with cis-pinosylvin (MOL009048), PIT (MOL00284), 4′-methylpinosylvin (MOL009362), pterostilbene (MOL011980), cis-resveratrol (MOL001229), dihydroresveratrol (MOL009782), cudranin (MOL012688), 5-[(Z)-2-(3, 4-dihydroxyphenyl) vinyl] resorcinol (MOL004570), and 5-[(Z)-2-(3-hydroxy-4-methoxy-phenyl) vinyl] resorcinol (MOL002262). These results indicate that resveratrol and *α*-viniferin were common stilbene compounds. In limited studies, stilbenes were proved to be highly related to targets of leukemia treatments, for example, pterostilbene, a 3′, 5′-dimethoxy-resveratrol interacted with 25 targets, including MAPK14, CDK2, and HSP90AA1, which are highly related to leukemia. Hsiao et al. have reported that pterostilbene induces apoptosis in HL-60 cells by MAPK-mediated regulation of the mitochondrial apoptotic pathway while also causing cell cycle arrest in the G0/G1 phase and inhibiting CDK2 expression [32]. In the present study, resveratrol and *α*-viniferin were found to be associated with antitumor targets, including Bcl-2, caspase-3, 8, and 9, SIRT1, and others. Taken together, these results show that *α*-viniferin, a resveratrol dimer, is predicted to be highly related to targets of leukemia, which are closely associated with its structure. Thus, the mechanism of leukemia treatment using *α*-viniferin needs to be clarified.

### 3.5. *α*-Viniferin Inhibits Cell Proliferation in K562 Cells

**Figure 4:**
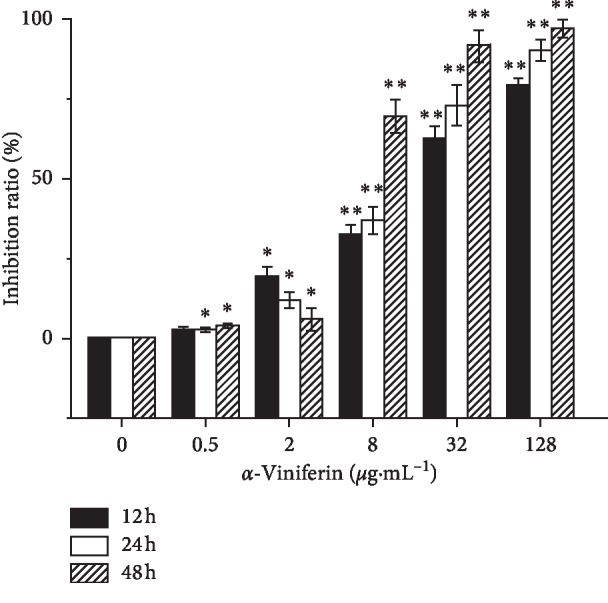
Effect of *α*-viniferin (obtained from BioPha Co., Ltd. Yunnan, China; cat no. BBP00220) on the cell inhibition ratio of K562 cells. Cells were treated with different concentrations of *α*-viniferin for 12, 24, and 48 h. Cell inhibition ratio was detected by the MTT assay. The results are the means of three independent experiments. ^*∗∗*^*P* < 0.01; ^*∗*^*P* < 0.01 vs. control.

Stilbene compounds were found to be capable of having high sensitivity towards leukemia cells by network analysis. Through literature mining in cancer cell, antiproliferative activities were shown with IC_50_ values of 3 to 4 *μ*g·mL^−1^ (HL-60) [33], 7 to 23 *μ*g·mL^−1^ (MCF-7) [34], 30 to 35 *μ*g·mL^−1^ (HepG2), 32 to 35 *μ*g·mL^−1^ (HepG2) [35], 50 *μ*g·mL^−1^ (A549) [33], and 37 to 45 *μ*g·mL^−1^ (WRL-68) [36]. Why the low concentration of the stilbene compounds had a high sensitivity to leukemia cells? Previously, it has been reported that resveratrol can cause cell apoptosis in various kinds of cancer cell lines, such as leukemia cells [37], breast carcinoma cells [38], and hepatoma cells [39]. According to the National Cancer Institute Developmental Therapeutics Program records (NSC 655524) (https://dtp.cancer.gov/dtpstandard/dwindex/index.jsp), *α*-viniferin is more sensitive to K562 cells in 32 candidate compounds by comparing GI_50_ and one dose growth percent (Supplemental [Supplementary-material supplementary-material-1]). Resveratrol had remarkable cytotoxic effects and induced apoptosis in K562 cells, and IC_50_ value is 9 *μ*g·mL^−1^. There were few records about *α*-viniferin inhibiting K562 cell growth. To verify the underlying antitumor activity of *α*-viniferin, MTT assay was used to detect the cytotoxic activity of different concentrations of *α*-viniferin ranging from 0.5 to 128 *μ*g·mL^−1^ on K562 cells. The results revealed that *α*-viniferin significantly inhibited the proliferation of K562 cells in both dose- and time-dependent manners (Figure 4). The inhibition rates sharply increased at the concentration of *α*-viniferin from 8 to 128 *μ*g·mL^−1^ at 12, 24, and 48 h. We observed *α*-viniferin inhibits K562 cell growth IC_50_ values of 13.61 *μ*g·mL^−1^ at 24 h. Compared with resveratrol, there were few records about *α*-viniferin, a resveratrol trimer, inhibiting K562 cell growth. But the effect of *α*-viniferin is not simply triple of resveratrol; it is probably due to another way of inhibiting the growth of K562 cells. We observed high-dose *α*-viniferin (32 to 128 *μ*g·mL^−1^) caused serious cell death, cell fragmentation, and nuclei lysis. Thus, we chose a comparatively lower concentration range from 0 to 32 *μ*g·mL^−1^ at 24 h for the investigation of mechanisms relating to the inhibitory effects of *α*-viniferin on K562 cell proliferation. In order to verify the selectivity of *α*-viniferin on leukemia cells, peripheral blood mononuclear cells (PBMCs) from healthy C57BL/6 mice were isolated and incubated with Con A or different concentrations of *α*-viniferin for 24 h and 48 h to detect cell viability. In total, *α*-viniferin promoted PBMC proliferation, but the proliferation effect of PBMC was observed only in 32 *μ*g·mL^−1^ with significance (Supplemental [Supplementary-material supplementary-material-1]). We also noticed that *α*-viniferin inhibits the proliferation of K562 cells by reducing the expression of the BCR-ABL protein (Supplemental [Supplementary-material supplementary-material-1]).

### 3.6. *α*-Viniferin Induces Proapoptotic Morphology in K562 Cells

**Figure 5:**
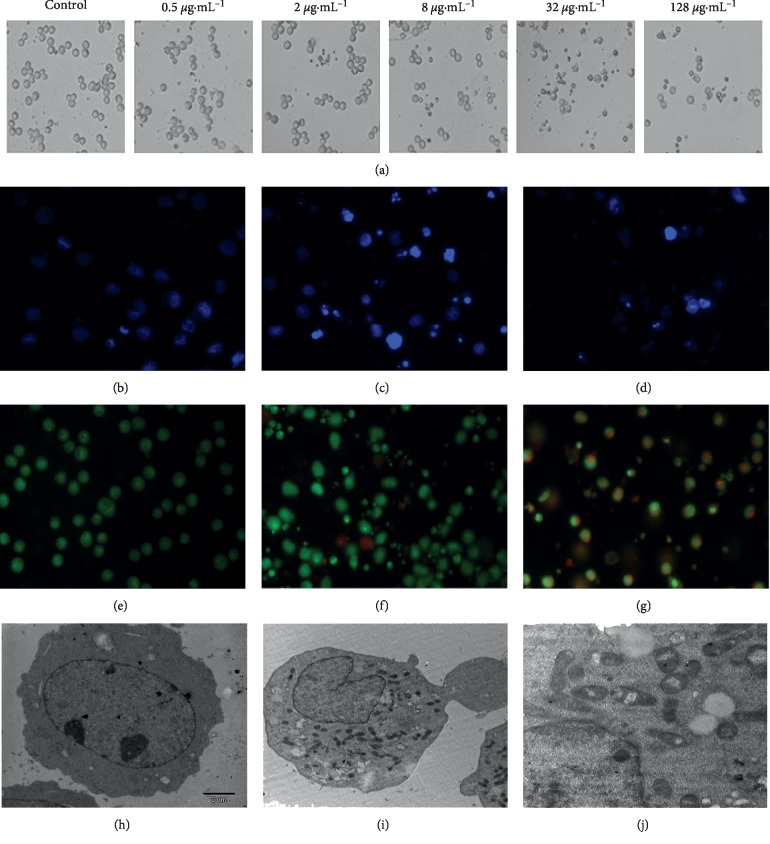
The images in (a) show the morphological changes of K562 cells treated with 0–128 *μ*g·mL^−1^*α*-viniferin for 24 h (magnified by 200x); the images in (b–d) show the morphological changes in the k562 cell nucleus with Hoechst staining; (b, c) K562 cells treated with 0∼32 *μ*g·mL^−1^*α*-viniferin for 24 h (magnified by 200x); (d) K562 cells treated with H_2_O_2_ for 24 h (magnified by 200x); the images in (e–g) show differences in k562 cell apoptosis and necrosis with AO/EB staining; (e, f) K562 cells treated with 0∼32 *μ*g·mL^−1^*α*-viniferin for 24 h (magnified by 200x); (g) K562 cells treated with H_2_O_2_ for 24 h (magnified by 200x); the images show ultrastructural features of a representative control cell (h) and the morphological features of apoptosis in K562 cells treated with 32 *μ*g·mL^−1^ α-viniferin (i, j) by electron microscopy; (h) K562 cells without *α*-viniferin treatment (magnified by 5,000x). K562 cells treated with 32 *μ*g·mL^−1^ for 24 h; (i) magnified by 6,000x; (j) magnified by 12,000x).

As shown in the microscopy images in Figure 5(a), following exposure to 0 to 128 *μ*g·mL^−1^*α*-viniferin for 24 h, K562 cells exhibited apoptotic morphological features, including distortion and disruption, all of which showed no difference with the cell proliferation assay results. In Figures 5(b)–5(d), Hoechst-stained control cells showed a circular nucleus containing diffuse Hoechst chromatin staining. After *α*-viniferin treatment, the cell nuclei became granular, which is consistent with the results of apoptosis-positive drug H_2_O_2_. In addition, cells stained with AO/EB appeared as bright green arcs and had condensed yellow/orange nuclei during the early stage and the late stage of apoptosis; however, no necrotic cells were observed (Figures 5(e)–5(g)). Besides, the ultrastructural analysis further proved the absence of apoptotic cells in the control group (Figure 5(h)). But apoptotic bodies were spotted in K562 cells treated with 32 *μ*g·mL^−1^*α*-viniferin for 24 h (Figures 5(i) and 5(j)).

### 3.7. Flow Cytometric Assay on Apoptosis

**Figure 6:**
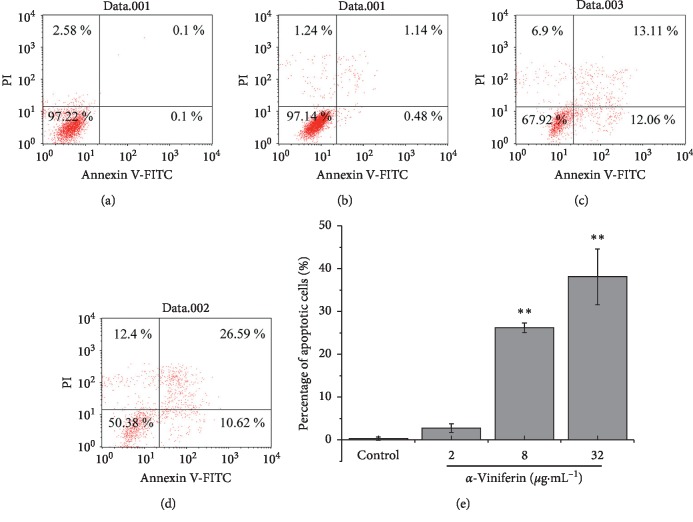
*α*-Viniferin induced early apoptosis of K562 cells in a dose-dependent manner. K562 cells were cultured with 0, 2, 8, and 32 *μ*g·mL^−1^ (a∼d) *α*-viniferin for 24 h analyzed by flow cytometry; relative quantitative analysis of apoptosis cells for 24 h (e); the results and error bars are the means of three independent experiments. ^*∗∗*^*P* < 0.01; ^*∗*^*P* < 0.05.

After *α*-viniferin treatment, K562 cells were analyzed by Annexin V-PI staining analysis to evaluate apoptotic cell death. Despite the pronounced concentration-dependent cell death that was observed in the cytotoxicity assay, apoptotic cell death was pronounced only at a high concentration of *α*-viniferin (Figure 6). To gain insights into the antileukemic effect of *α*-viniferin, we performed FITC Annexin V and PI staining of K562 cells. Cells treated for 24 h with *α*-viniferin revealed marked flow cytometry findings, including a few cells appearing in the second quadrant (FITC Annexin V-negative and PI-positive), indicating that necrosis rarely occurred in the drug-treated cells. The finding of drug-induced cell apoptosis, indicated by cells moving from the third (FITC Annexin V- and PI-negative) to the first quadrant (FITC Annexin V- and PI-positive) and fourth quadrants (FITC Annexin V-positive and PI-negative), suggested that apoptosis was induced by the treatment.

### 3.8. Mechanism of *α*-Viniferin-Induced Apoptosis

**Figure 7:**
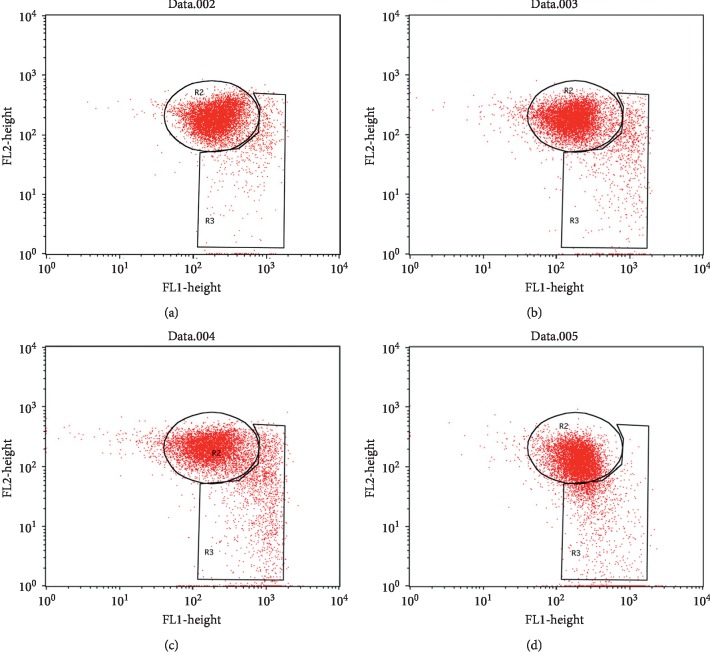
*α*-Viniferin induces apoptosis in K562 cells by reducing mitochondrial membrane potential. K562 cells were cultured with 0∼32 *μ*g·mL^−1^*α*-viniferin for 24 h and stained with JC-1. (a) Control. (b) 2 *μ*g·mL^−1^. (c) 8 *μ*g·mL^−1^. (d) 32 *μ*g·mL^−1^.

To verify the potential mechanism by which *α*-viniferin induces apoptosis, we detected changes in the mitochondrial membrane potential involved in apoptosis. To explore the effects of *α*-viniferin on mitochondrial membrane potential, JC-1-stained K562 cells were analyzed by flow cytometry, which were cultured in the presence of different drug concentrations for 24 h (Figure 7). Compared with untreated cells, mitochondrial membrane potential decreases in a dose-dependent manner. This result showed that *α*-viniferin disrupts the mitochondrial membrane potential leading to cytosolic accumulation of monomeric JC-1, activating the intrinsic pathway to indicate apoptosis.

**Figure 8:**
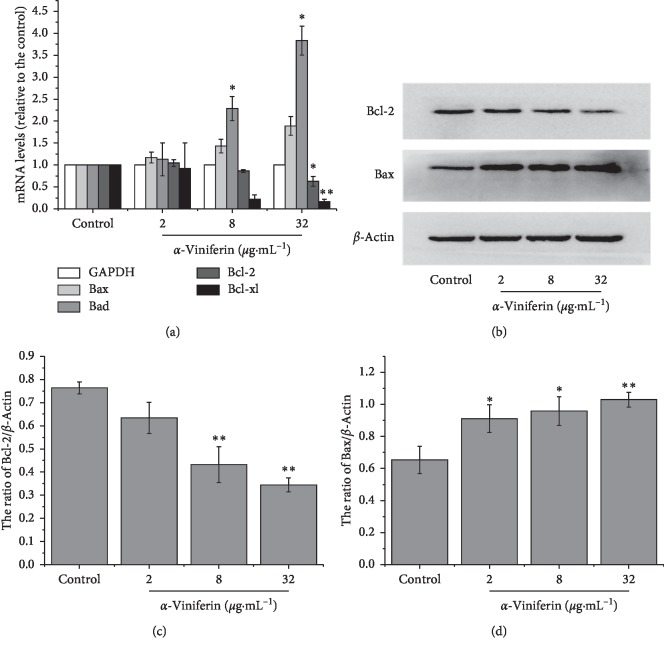
Evaluation of mRNA and protein expression of the Bcl-2 family. K562 cells are cultured with 0∼32 *μ*g·mL^−1^*α*-viniferin for 24 h. (a) Evaluation of mRNA expression of Bcl-2 family by quantitative real-time RT-PCR (qRT-PCR); (b) Evaluation of protein expression of Bcl-2 family by western blotting; relative quantitative analysis of protein expression (c, d). Bcl-2 and Bcl-xL expression decreased, and Bad and Bax expression increased. The results and error bars are the means of three independent experiments. ^*∗∗*^*P* < 0.01; ^*∗*^*P* < 0.05.

The mRNA levels of Bad, Bax, Bcl-2, and Bcl-xL were measured in *α*-viniferin-induced and control K562 cells by quantitative real-time RT-PCR (qRT-PCR). As shown in Figure 8(a), when *α*-viniferin was added, the mRNA levels of apoptosis-promoting genes were upregulated with increasing drug concentrations. In contrast, antiapoptosis mRNA expression levels were significantly downregulated. However, the results of Bcl-2 and Bax are not very significant compared to Bcl-xL and Bad. To further verify the observed changes in Bcl-2 family underlying *α*-viniferin-induced apoptosis, the protein levels of Bcl-2 and Bax were measured by western blotting. Compared with the control group, Bcl-2 expression decreased, and Bax expression increased after treated with *α*-viniferin for 24 h (Figures 8(b)–8(d)). And the mRNA levels of caspase-9 were upregulated in a dose-dependent manner measured by RT-PCR (Supplemental [Supplementary-material supplementary-material-1]).

## 4. Discussion

We found changes in the apoptosis pathway after drug treatment, and the expression of Bcl-2 family changed significantly. These findings suggest that stilbene compounds do not rely on toxicity to kill cancer cells; instead, cancer cells are killed through mitochondrial pathway interference of their growth and induction of apoptosis. Scientists working in the field of apoptosis agree that programmed cell death occurs via mitochondrial damage. For example, a recent study has found that resveratrol is a tumor suppressor compound in grapes that induces apoptosis through a mitochondrial pathway regulated by Bcl-2 [40]. Studies have shown that resveratrol induces apoptosis in different acute lymphoblastic leukemia cells by depolarizing mitochondrial membranes and activating caspase-9 [41]. Wieder et al. reported that a hydroxylation analogue of resveratrol, named piceatannol, could induce apoptosis in the lymphoma cell line BJAB and primary leukemic lymphoblasts [42]. Resveratrol-mediated stimulation of C7H2 cells has been shown to lead to the production of reactive oxygen species (ROS), which is remarkably reduced by Bcl-2 [32]. Another study has shown that resveratrol induces apoptosis in K562 cells by reducing mitochondrial membrane potential [43]. This study observed the same phenomenon for 32 *μ*g·mL^−1^*α*-viniferin treated with K562 cells for 24 h.

Lower cytotoxicity and higher protective potency appear to be common characteristics of natural stilbenes. However, *α*-viniferin may play a role in plant disease resistance, human health, and other diseases. A large number of Chinese medicines analyzed by systems pharmacology have been found to contain *α*-viniferin and to exhibit an effect on leukemia cells. According to the National Cancer Institute Developmental Therapeutics Program records (NSC 655524) (https://dtp.cancer.gov/dtpstandard/dwindex/index.jsp), leukemia and central nervous system cell lines are sensitive to *α*-viniferin treatments *in vitro*. We used the Comparative Toxicogenomics Database to evaluate the specificity of *α*-viniferin against different diseases according to inferred scores [44]. Among the top 100 inferred diseases related to *α*-viniferin, cancer and cardiovascular disease were highly correlated with *α*-viniferin. These results indicate the possible proapoptotic activity of *α*-viniferin for potential application in treating cancer, whose effects were observed in our work. However, the antitumor mechanism of *α*-viniferin is likely greater than this single effect. Its role in cell cycle arrest, autophagy, and other functions must be investigated further.

## 5. Conclusions

In summary, our study is the first systems pharmacology investigation of the treatment of CML by TCM. We generated 118 bioactive compounds from 11 herbs within four dialectical therapy groups and 141 predicted targets. Our data showed that *α*-viniferin, a natural stilbene, was found to be highly related to these targets. And it has lower cytotoxicity and high sensitivity to K562 cells. We found *α*-viniferin could induce early apoptosis on K562 cells, especially considering the Bcl-2 family of proteins in regulating K562 cell apoptosis through unique modulation of the mitochondrial pathway. *α*-Viniferin caused mitochondrial damage and increased Bcl-2 family proapoptotic protein activity, leading to apoptosis. Thus, we hypothesize that *α*-viniferin merits additional experimental and clinical research in the treatment of CML.

## Figures and Tables

**Table 1:** The ADME properties *in silico* of 32 candidate compounds via admetSAR prediction.

Compounds	Absorption		Metabolism (CYP450 isoform inhibitor)					
	HIA	Caco-2	1A2	2C9	2D6	2C19	3A4	Score
Majudin	0.9933	0.6835	0.9774	0.8257	0.8931	0.9296	0.7959	3.48407919
Bergapten	0.9964	0.5379	0.9107	0.8949	0.8931	0.8994	0.8123	3.47147818
Sesamin	1	0.5774	0.9106	0.8949	0.8932	0.8994	0.796	3.46096872
Suchilactone	0.9884	0.7833	0.6598	0.9399	0.8096	0.9801	0.9591	3.39143188
Isorhamnetin	0.9783	0.8866	0.9218	0.756	0.6993	0.8648	0.7348	3.12557861
Ammidin	0.9962	0.5187	0.8503	0.7199	0.7115	0.8977	0.7497	3.08492296
8-Geranoxy-5-methoxypsoralen	0.9924	0.5556	0.8058	0.7084	0.788	0.8398	0.7848	3.0808701
Cnidilin	0.9925	0.5243	0.8169	0.5477	0.641	0.8984	0.6951	2.82470425
Naringenin	0.967	0.7533	0.9106	0.8949	−0.7463	0.8994	0.8988	2.12533827
Genkwanin	0.9921	0.929	0.9378	0.9385	−0.8253	0.9012	0.7232	2.00309141
Diosmetin	0.9783	0.8866	0.9218	0.756	−0.6993	0.8648	0.7348	1.92963575
Chrysoeriol	0.9783	0.8866	0.9218	0.756	−0.6993	0.8648	0.7348	1.92963575
Kaempferide	0.9783	0.8866	0.9218	0.756	−0.6993	0.8648	0.7348	1.92963575
Syringetin	0.9719	0.8896	0.8668	0.6258	−0.6249	0.8187	0.7817	1.83717389
4′-Methylpinosylvin	0.9968	0.9191	0.9614	0.903	−0.8899	0.886	0.5	1.78240889
Dehydrodieugenol	0.9721	0.8004	0.744	0.797	−0.7521	0.9261	0.6365	1.75847413
Moracin M	0.9964	0.6043	0.9061	0.7842	−0.8215	0.7031	0.804	1.74936332
Kaempferol	0.9855	0.7447	0.9108	0.8948	−0.9083	0.6434	0.7241	1.66803093
Morin	0.9855	0.7447	0.9108	0.8948	−0.9083	0.6434	0.7241	1.66803093
Notoptol	0.9762	0.5537	0.7233	0.7758	−0.6654	0.7588	0.6368	1.66419893
Dihydroresveratrol	0.9503	0.8604	0.7749	0.742	−0.8326	0.8434	0.7201	1.65802923
Cis-resveratrol	0.9952	0.8915	0.9106	0.7068	−0.9226	0.8052	0.7539	1.65443638
Cis-pinosylvin	0.9948	0.9014	0.9177	0.6648	−0.9171	0.8322	0.6678	1.5974595
Oxysanguinarine	0.9841	0.7216	0.8373	−0.8557	0.5126	0.7982	0.669	1.50874759
*α*-Viniferin	1	0.6216	0.9277	0.9497	−0.7239	0.8042	−0.6358	1.13587144
(1H,3H-Furo[3,4-c]furan-3a(4H)-ol, dihydro-1,4-bis(4-hydroxy-3-methoxyphenyl)-, (1R,3aS,4S,6aR)-)	0.5176	0.6408	0.6819	0.5427	−0.8266	0.5876	0.542	1.10669817
Isoflavone	1	0.8727	0.9662	0.7441	−0.9436	0.9213	−0.6675	0.88838768
4,7-Dihydroxy-5-methoxyl-6-methyl-8-formyl-flavan	0.9889	0.9077	0.7694	0.565	−0.6802	0.7659	−0.6223	0.71368052
6,8-Dihydroxy-7-methoxyxanthone	0.9306	0.9044	0.9724	−0.6549	−0.6476	0.6923	0.6321	0.67863562
(−)-Medioresinol	0.9944	0.763	−0.5381	0.6912	−0.8313	0.7443	0.5296	0.34602068
(+)-Medioresinol	0.9944	0.763	−0.5381	0.6912	−0.8313	0.7443	0.5296	0.34602068
Torachrysone	0.9803	0.92	0.9469	−0.7326	−0.8119	0.5056	0.5459	0.2491008

## Data Availability

All data generated or analyzed during this study are included in this published article and its supplementary information files.

## Abbreviations

ADMET: Drug absorption, distribution, metabolism, excretion, and toxicity
Bad: Bcl2-associated agonist of cell death
Bax: Apoptosis regulator BAX
Bcl-2: Apoptosis regulator Bcl-2
Bcl-xL: Antiapoptotic regulator Bcl-xL
Caco-2: Caco-2 permeability
CDK2: Cyclin-dependent kinase 2
CML: Chronic myeloid leukemia
HIA: Human intestinal absorption
HSP90AA1: Heat-shock protein HSP 90-alpha
IC_50_: Half maximal inhibitory concentration
K562: Human chronic myeloid leukemia cell
MAPK14: Mitogen-activated protein kinase 14
MTT: 3-(4, 5-Dimethyl-thiazol-2-yl)-2,5-diphenyl tetrazolium bromide
PBMC: Peripheral blood mononuclear cells
TCM: Traditional Chinese medicine.

## Supplementary Materials

Supplementary Materials Supplemental Table 1: TCM prescriptions for CML. Supplemental Table 2: full ADME properties *in silico* of 121 candidate compounds via admetSAR Prediction. Supplemental Table 3: intersection of CML targets and prediction Targets. Supplemental Table 4: literature mining of 32 active compounds. Supplemental Table 5: NCI Cancer Screen Data of 32 candidate compounds on K562 cells. Supplemental Figure 1: effects of *α*-viniferin on peripheral blood mononuclear cells (PBMC) proliferation. Supplemental Figure 2: evaluation of *α*-viniferin on the protein expression level of BCR-ABL. Supplemental Figure 3: evaluation of *α*-viniferin on the mRNA level of caspase-9.
