# Supplementary material for: System Prediction and Validation of TCM for Chronic Myeloid Leukemia Treatment from the Perspective of Low-Toxicity Chemotherapy: A Stilbene α-Viniferin Has a Proapoptotic Effect on K562 Cells via the Mitochondrial Pathway
Source: Evid Based Complement Alternat Med. 2020 Feb 10;2020:1986962. doi: 10.1155/2020/1986962 (PMC7035520; doi:10.1155/2020/1986962)
Supplement: Supplementary Materials — Supplemental Table 1: TCM prescriptions for CML. Supplemental Table 2: full ADME properties in silico of 121 candidate compounds via admetSAR Prediction. Supplemental Table 3: intersection of CML targets and prediction Targets. Supplemental Table 4: literature mining of 32 active compounds. Supplemental Table 5: NCI Cancer Screen Data of 32 candidate compounds on K562 cells. Supplemental Figure 1: effects of α-viniferin on peripheral blood mononuclear cells (PBMC) proliferation. Supplemental Figure 2: evaluation of α-viniferin on the protein expression level of BCR-ABL. Supplemental Figure 3: evaluation of α-viniferin on the mRNA level of caspase-9. [file 1986962.f1.zip › Supplementary Materials/Supplemental Figure 1.pdf]

Method

1. Isolate Peripheral blood mononuclear cells

Peripheral blood mononuclear cells (PBMC) were isolated from healthy C57BL/6 mice with Mouse Peripheral Blood Mononuclear Cell Isolation Kit (P6340, Solarbio). Briefly, heparinized blood was mixed with an equal volume of Whole blood dilution. Samples were layered over 5 mL Ficoll-Histopaque in a 15 mL glass centrifuge tube. After centrifuging at 2500 rpm for 30 min at room temperature, the interface layer of PBMC was carefully sucked out and washed twice with cell washing solution at 1000 rpm for 10 min.

2. PBMC identification

2.1. Wright-Giemsa staining

After the cell smear is dried, add 0.8 mL of Wright-Giemsa A solution to the slide to cover the whole smear with the staining solution for 1 minute, and then add the B solution to the A solution (the amount of the B solution is 2-3 times of solution A), blow out the breeze with the ear wash ball to make the liquid surface ripple, so that the two liquids are fully mixed. After dyeing for 3-10 min, wash directly with water, dry, and take a photo with a microscope.

2.2. Flow cytometry

Take an appropriate amount of peripheral blood mononuclear cells in a test tube, mix with the sheath fluid, and observe the cell group by FSC and SSC on the Flow cytometry.

3. Assessment of PBMC Proliferation Ability

The PBMC were cultured in RPMI-1640 containing 10% fetal bovine serum and 100 U·mL^{-1} each of penicillin and streptomycin in a humidified atmosphere maintained at 37˚C and 5% CO_2. The cells were inoculated with 4×10^4 /mL per well in a 96-well culture plate, and the volume of the culture medium was 200 μL. Different concentrations of α-viniferin or 1 μg·mL^{-1} Con A were added, and cultured at 37 °C for 24 and 48 h. Then, the plates were centrifuged at approximately 800 ×g for 5 minutes and discarded the supernatant. Finally, 100 μL RPMI 1640 containing 10% CCK8 was added into each well to detect the OD values and to calculate the PBMC proliferation rate.

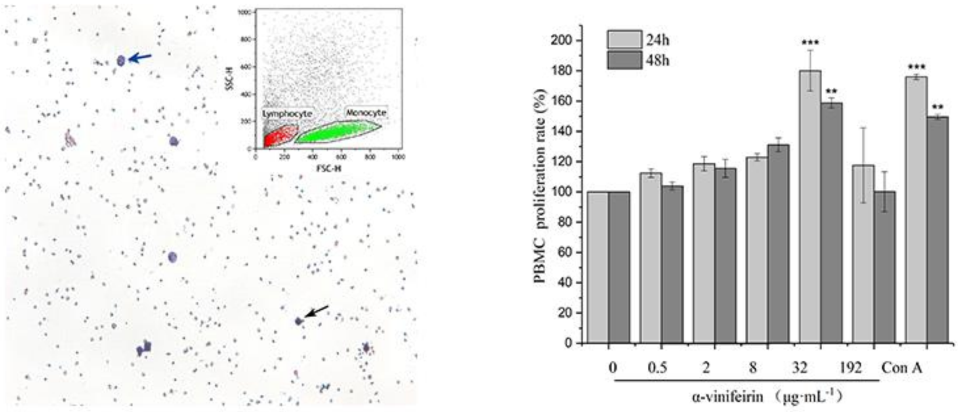

a b

Supplemental Figure 1： Effect of α-viniferin on the cell proliferation rate of peripheral blood mononuclear cells (PBMC). We isolated peripheral blood mononuclear cells (PBMC) from healthy C57BL/6 mice. a. We have identified PBMC by Wright-Giemsa staining and Flow

cytometry. Blue arrows: monocytes; black arrows: lymphocytes. b. PBMC were incubated with different concentration of α-viniferin or 1 μg·mL^{-1} Con A for 24 h, 48h to study the cell proliferation, cell proliferation rate was detected by the CCK8 assay. The results were the means of three independent experiments(***) P<0.001, (**) P<0.01, (*) P<0.05 vs control.
