# Supplementary material for: System Prediction and Validation of TCM for Chronic Myeloid Leukemia Treatment from the Perspective of Low-Toxicity Chemotherapy: A Stilbene α-Viniferin Has a Proapoptotic Effect on K562 Cells via the Mitochondrial Pathway
Source: Evid Based Complement Alternat Med. 2020 Feb 10;2020:1986962. doi: 10.1155/2020/1986962 (PMC7035520; doi:10.1155/2020/1986962)
Supplement: Supplementary Materials — Supplemental Table 1: TCM prescriptions for CML. Supplemental Table 2: full ADME properties in silico of 121 candidate compounds via admetSAR Prediction. Supplemental Table 3: intersection of CML targets and prediction Targets. Supplemental Table 4: literature mining of 32 active compounds. Supplemental Table 5: NCI Cancer Screen Data of 32 candidate compounds on K562 cells. Supplemental Figure 1: effects of α-viniferin on peripheral blood mononuclear cells (PBMC) proliferation. Supplemental Figure 2: evaluation of α-viniferin on the protein expression level of BCR-ABL. Supplemental Figure 3: evaluation of α-viniferin on the mRNA level of caspase-9. [file 1986962.f1.zip › Supplementary Materials/Supplemental Figure 3.pdf]

Method

1. Evaluation of Caspase-9 gene expression

Detection of Caspase-9 gene expression using quantitative RT-PCR, which was performed using PCR Master Mix (Sangong Co.), cDNA template, and each primer. The RT-PCR products were semi-quantitatively assessed by 0.5% agarose gel electrophoresis using a GelDOC analysis system (Bio-Rad).

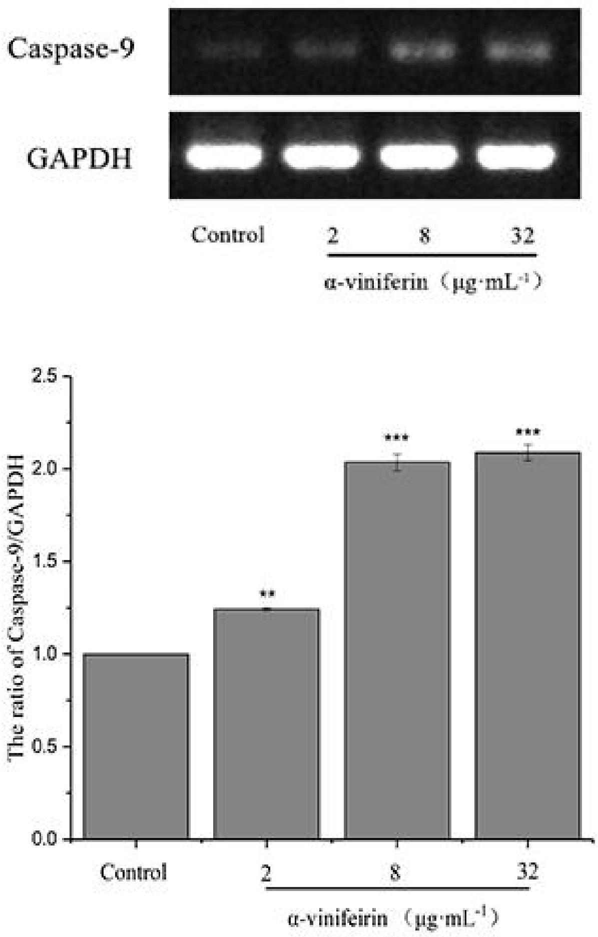

Supplemental Figure 3: Evaluation of α-viniferin on the mRNA expression of Caspase-9. K562 cells were cultured with 0~32 $\mu g \cdot mL^{-1}$ α-viniferin for 24 h. Evaluation of mRNA expression of Caspase-9 by RT-PCR and performing relative quantitative analysis of mRNA expression (***) $P<0.001$, (**) $P<0.01$, (*) $P<0.05$ vs control.
