# Supplementary material for: System Prediction and Validation of TCM for Chronic Myeloid Leukemia Treatment from the Perspective of Low-Toxicity Chemotherapy: A Stilbene α-Viniferin Has a Proapoptotic Effect on K562 Cells via the Mitochondrial Pathway
Source: Evid Based Complement Alternat Med. 2020 Feb 10;2020:1986962. doi: 10.1155/2020/1986962 (PMC7035520; doi:10.1155/2020/1986962)
Supplement: Supplementary Materials — Supplemental Table 1: TCM prescriptions for CML. Supplemental Table 2: full ADME properties in silico of 121 candidate compounds via admetSAR Prediction. Supplemental Table 3: intersection of CML targets and prediction Targets. Supplemental Table 4: literature mining of 32 active compounds. Supplemental Table 5: NCI Cancer Screen Data of 32 candidate compounds on K562 cells. Supplemental Figure 1: effects of α-viniferin on peripheral blood mononuclear cells (PBMC) proliferation. Supplemental Figure 2: evaluation of α-viniferin on the protein expression level of BCR-ABL. Supplemental Figure 3: evaluation of α-viniferin on the mRNA level of caspase-9. [file 1986962.f1.zip › Supplementary Materials/Supplemental Table-1.pdf]

Supplemental Table-1. TCM prescriptions for CML

	source	Formula name	Formula composition
	《备急千金要方》卷六	戎盐丸	*Scutellaria baicalensis* Georgi. Dried Root、*Phellodendron amurense Rupr*. Dried Root、*Rheum palmatum L. Dried,Root* C.A.Mey. Dried Root、*Glycyrrhiza uralensis* Fisch. Dried Root.
	《伤寒六书》卷三	黄龙汤	*Glycyrrhiza uralensis* Fisch. Dried Root、*Nairii* Sulfas、*Citrus aurantium* L.　Dry yung fruit、*Magnolia officinalis Rehder* & E.H.Wilson， Dry branches, root bark、*Panax ginseng* C.A.Mey. Dried Root、*Angelica sinensis* (Oliv.) Diels. Dried Root、*Glycyrrhiza uralensis* Fisch. Dried Root.
Heat clearing and blood cooling	蔡永良方	泽黄英土汤	*Lycopus lucidus* var. hirtus Regel、*Rhinoceros unicornis* L, Dried Root（Brewed with wine）、*Taraxacum mongolicum* Hand.-Mazz. Dried Root、*Smilax glabra* Roxb. Dried Root、*Lycopus lucidus* Turcz. ex Benth. Dried Root、*Isatis indigotica* Fortune ex Lindl. Dried Root、*Artemisia scoparia* Waldst. & Kitam. Dried aboveground.
	黄文章方	虎杖煎剂	*Lycopus lucidus* Turcz. ex Benth. Dried Root、*Isatis indigotica* Fortune ex Lindl. Dried Root、*Taraxacum mongolicum* Hand.-Mazz. Dried Root、Artemisia scoparia Waldst. & Kitam. Dried aboveground、*Citrus reticulata* Blanco, Dried fruit peel.
	蒋森方	虎杖解毒汤	*Lycopus lucidus* Turcz. ex Benth. Dried Root、Isatis indigotica Fortune ex Lindl. Dried Root、Paeonia lactiflora PalL, Dry root、Cryptotympanapustulata Fabricius、*Moutan officinalis* (L.) Lindl. & Paxton, cortex.

《万病回春》卷四	清火滋阴汤	*Asparagus cochinchinensis*（Lour.） Merr.、Ophiopogon japonicus （L.f） Ker-GawL、*Rehmannia glutinosa* (Gaertn.) DC. Dry root、*Moutan officinali*s (L.) Lindl. & Paxton, cortex、*Paeonia lactiflor*a PalL, Dry root、*Gardenia jasminoides* J.Ellis, Dried nutlet、*Dioscotea opposita* Thunb.、*Cornus officinalis* Sieb.et Zucc.Dry yung fruit
《医学入门》卷八	柴胡抑肝汤	*Bupleurum chinense* DC. Dried Root、Paeonia lactiflora PalL, Dry root、*Moutan officinalis* (L.) Lindl. & Paxton, cortex、*Citrus reticulata Blanco,Dried young fruit peel*、Forsythia suspensa （Thunb.） Vahl, Dried fruit、Lycium chinense Mil, Dried root bark、Cyperus rotundus L., Dry rhizome、Atractylodes lancea （Thunb.）DC, Dry rhizome、Gardenia jasminoides J.Ellis, Dried fruit、Ligusticum acuminatum Franch. Dry rhizome、*Glycyrrhiza uralensis* Fisch. Dried Root
《活人方》卷二	结阴丸	*Rehmannia glutinosa* (Gaertn.) DC. Dry root、Paeonia lactiflora Pall. Dried root、Dioscotea opposita Thunb.、SophorajaponicaL. Dry flower bud、Coptis chinensis Franch, Dry rhizome、Schizonepeta tenuifolia (Benth.) Briq. Dried aboveground、Rubia cordifolia L. Dry rhizome and root、*Sanguisorba officinalis* L. Dried root、*Prunus alabamensis* C.Mohr, Dried pulp、Cimicifuga heracleifolia Kom. Dried rhizome、*Platycladus orientalis* （L.） Franco, Dry branches slightly and leaves
钟磊方	清金养血汤	*Ligusticum acuminatum* Franch. Dry rhizome、*Angelica sinensis* (Oliv.) Diels. Dried Root、*Paeonia lactiflora* Pall. Dried root、*Cyperus rotundus* L., Dry rhizome、*Ophiopogon japonicus*（L.f） Ker-GawL、*Moutan officinalis* (L.) Lindl. & Paxton, cortex、*Cyathula achyranthoides* (Kunth) Moq. Dry rhizome、*Lycium chinense* Mil,

		Dried root bark、*Rehmannia glutinosa* (Gaertn.) DC. Dry root、*Schisandra arisanensis* Hayata, Dry ripe fruit、*Glycyrrhiza uralensis* Fisch. Dried Root, Honeyed
《言庚孚医疗经验集》	抗原汤	*Angelica sinensis* (Oliv.) Diels. Dried Root、*Paeonia lactiflora* Pall. Dried root、*Cyathula achyranthoides* (Kunth) Moq. Dry rhizome、*Bupleurum chinense* DC. Dried Root、*Poria cocos*（Schw.）Wolf, Dried Root、*Lycopus lucidus* Turcz. ex Benth. Dried Root、*Artemisia scoparia* Waldst. & Kitam. Dried aboveground、*Hedyotis diffusa* var. extensa (Hook.f.) R.Dutta, Dried aboveground、*Glycyrrhiza uralensis* Fisch. Dried Root
《万病回春》卷六	达原败毒散	*Paeonia lactiflora* Pall. Dried root、*Arecacatechu* L. Dry mature seeds、*Notopterygium incisum* K.C.Ting ex H.T.Chang, Dry rhizome and root、*Angelica pubescens* Maxim.Dried root、*Bupleurum chinense* DC. Dried Root、*Stellaria dichotoma* L. Dried Root、*Platycodon grandiflorum*（Jacq.）A.DC. Dried Root、*Magnolia officinalis* Rehder & E.H.Wilson， Dry branches, root bark、*Citrus aurantium* L. Dry young fruit、*Poria cocos*（Schw.）Wolf, Dried Root、*Moutan officinalis* (L.) Lindl. & Paxton, cortex、*Mentha haplocalyx* Briq. Dry leaf、*Amomum tsao-ko* Crevost et Lemaire, Dried nutlet、*Achyranthes bidentata* Blume, Dried root.
《金匮要略》	大黄蛰虫丸	*Glycyrrhiza uralensis* Fisch. Dried Root、*Nairii Sulfas*、*Eupolyphaga sinensis* Walker、*Whitmania pigra* Whitman、*Tabanus bivittatus* Matsumura、Grub.

	《肘后方》卷八	屠苏酒	*Glycyrrhiza uralensis* Fisch. Dried Root、*Platycodon*、*grandiflorum*（Jacq.）A.DC. Dried Root、*Cyathula achyranthoides* (Kunth) Moq. Dry rhizome、*Aconitum abietetorum* W.T.Wang & L.Q.Li, Dried root、*Smilax china* L. Dry rhizome.
	《外台》卷十一引《近效方》	调中方	*Cimicifuga heracleifolia* Kom. Dried rhizome、*Scrophularia aequilabris* Tsoong, Dried root、*Glycyrrhiza uralensis* Fisch. Dried Root、*Dioscotea opposita* Thunb.、*Poria cocos*（Schw.）Wolf, Dried Root、*Ostrea gigas* Thunberg、*Rhaponticumuniflorum*（L.）DC. Dried root、*Citrus aurantium* L. Dried yung fruit、*Smilax china* L. Dry rhizome、*Coptis chinensis* Franch.,Dried Root.
Relieving poisoning and detoxifying	《华佗神医秘传》卷四	辟疫酒	*Glycyrrhiza uralensis* Fisch. Dried Root、*Cyathula achyranthoides* (Kunth) Moq. Dry rhizome、*Platycodon*、*grandiflorum*（Jacq.）A.DC. Dried Root、*Aconitum abietetorum* W.T.Wang & L.Q.Li, Dried root、*Smilax china* L. Dry rhizome
	《朱仁康临床经验集》	白疕二号方	*Smilax glabra* Roxb. Dried Root、*Lonicera acuminata* Wall. Dry stem、*Clematis chinensis* Osbeck, Dry rhizome and root、*Glycyrrhiza uralensis* Fisch. Dried Root、*Isatis indigotica* Fortune ex Lindl. Dried Root、*Sophora tonkinensis* Gagnep. Dry rhizome and root、*Dictamnus dasycarpus* Turcz. Dry bark
	《赵柄南临床经验集》	除湿解毒汤	*Dictamnus dasycarpus* Turcz. Dry bark、*Glycine arenaria* Tindale, Dry processed products after seed germination、*Coix lacryma-jobi* L. Dry mature seeds、*Gardenia jasminoides* J.Ellis, Dried nutlet、*Moutan officinalis* (L.) Lindl. & Paxton, cortex、*Lonicera japonica* Thunb. Dry flower buds or open flowers、*Corydalis bungeana* Turcz. Dry whole grass、*Forsythia suspensa*（Thunb.）Vahl, Dried fruit、*Akebia quinata*（Thunb.）Decne, Dry cane、*Glycyrrhiza uralensis* Fisch. Dried Root.

《全国中药成药处方集》（沈阳方）	花柳解毒丸	*Lonicera japonica* Thunb. Dry flower buds or open flowers、*Dictamnus dasycarpus* Turcz. Dry bark、*Coix lacryma-jobi* L. Dry mature seeds、*Akebia quinata*（Thunb.）Decne, Dry cane、*Smilax glabra* Roxb. Dried Root、*Saposhnikovia divaricata* （Turcz.） Schischk, Dried Root、*Chaenomeles speciosa* (Sweet) Nakai, Dry close to ripe fruit、*Gleditsia sinensis* Lam. Dry young fruit、*Angelica acutiloba* (Siebold & Zucc.) Kitag. Dry tail of the root、*Carthamus tinctorius* L. Dry flower、*Glycyrrhiza uralensis* Fisch. Dried Root.
僧焰如方	活络通脉清解汤	*Panax ginseng* C.A.Mey. Dried Root、*Angelica sinensis* (Oliv.) Diels. Dried Root、*Lonicera macranthoides* Hand.-Mazz. Dry stem、*Salvia miltiorrhiza* Bunge， Dry rhizome and root、*Ligusticum acuminatum* Franch. Dry rhizome、*Clematis chinensis* Osbeck, Dry rhizome and root、*Gleditsia sinensis* Lam. Dry spines、*Taraxacum mongolicum* Hand.-Mazz. Dried Root、*Smilax glabra* Roxb. Dried Root、*Glycyrrhiza uralensis* Fisch. Dried Root.
《实用中医外科学》	土茯苓合剂	*Smilax glabra* Roxb. Dried Root、*Lonicera japonica* Thunb. Dry flower buds or open flowers、*Clematis chinensis* Osbeck,Dry rhizome and root、*Dictamnus dasycarpus* Turcz. Dry bark、*Glycyrrhiza uralensis* Fisch. Dried Root、*Xanthium sibiricum* Patr. Dry fruit.
来春茂方	乙肝解毒汤	*Astragalus aaronii* (Eig) Zohary, Dried Root、*Lycopus lucidus* Turcz. ex Benth. Dried Root、*Hedyotis diffusa* var. extensa (Hook.f.) R.Dutta, Dried aboveground、*Scutellaria adenostegia* Briq. Dry whole grass、*Agrimonia pilosa* Ledeb. Dry aerial part、*Pyrola calliantha* Andres, Dry whole grass、*Smilax glabra* Roxb. Dried Root、*Bupleurum chinense* DC. Dried Root、*Curcuma longa* L, Dry root、*Angelica sinensis* (Oliv.) Diels. Dried Root、*Cinnamomum cassia* (L.) J.Presl,

			Dry twig、*Glycyrrhiza uralensis* Fisch. Dried Root.
	《原机启微》卷下	抑阳酒连散	*Rehmannia glutinosa* (Gaertn.) DC. Dry root、*Angelica pubescens* Maxim. Dried root、*Phellodendron amurense* Rupr. Dried Root、*Saposhnikovia divaricata* （Turcz.） Schischk, Dried Root、Anemarrhena asphodeloides Bge.、*Viburnum acerifolium* L. Dry mature fruit、*Peucedanum praeruptorum* Dunn, Dry root、*Angelica acutiloba* (Siebold & Zucc.) Kitag. Dry root、*Notopterygium incisum* K.C.Ting ex H.T.Chang, Dry rhizome and root、*Glycyrrhiza uralensis* Fisch. Dried Root、*Scutellaria baicalensis* Georgi. Dried Root、*Gardenia jasminoides* J.Ellis, Dried nutlet、*Coptis chinensis* Franch.,Dried Root、*Stephania tetrandra* S.Moore，Dried Root、
Eliminating wind and dispersing wet	《柳森可用方》	十七味药酒	*Ziziphusjujuba* Mill.var.spinosa（Bunge）HuexH.F.Chou, Dried nutlet、*Saiga tatarica* Linnaeus、*Dendrobium nobile* Lind, Dry stem、*Astragalus aaronii* (Eig) Zohary, Dried Root、*Notopterygium incisum* K.C.Ting ex H.T.Chang, Dry rhizome and root、*Rehmannia glutinosa* (Gaertn.) DC. Dry root、*Achyranthes bidentata* Blume, Dried root、*Cinnamomum cassia* (L.) J.Presl, Dry bark、*Poria cocos*（Schw.）Wolf, Dried Root、*Eucommia ulmoides* Oliv. Dry bark.
	李润民方	化瘀清散汤	*Bupleurum chinense* DC. Dried Root、*Pueraria alopecuroides* Craib, Dry root、*Salvia miltiorrhiza* Bunge, Dry rhizome and root、*Chrysanthemum morifolium* Ramat.Dry headed inflorescence、*Morus alba* L. Dry twig、*Moutan officinalis* (L.) Lindl. & Paxton, cortex、*Paeonia lactiflora* PalL, Dry root、*Carthamus tinctorius* L. Dry flower、*Pheretima aspergillum* E.Perrier、*Mentha haplocalyx* Briq. Dry leaf.

张海水方	疏风养血汤加减	*Gastro diaelata* Bl. Dry roots、*Hyriopsis cumingii* Lea, shell、*Paeonia lactiflora* Pall, Dry root、*Glycyrrhiza uralensis* Fisch. Dried Root、*Pueraria alopecuroides* Craib, Dry root、*Salvia miltiorrhiza* Bunge, Dry rhizome and root、*Unacaria rhynchophylla*（Miq.）Miq.ex Havil, Dry hooked stem、*Lonicera macranthoides* Hand.-Mazz. Dry stem、*Morus alba* L. Dry twig、*Gentiana macrophylla* Pall. Dry root、*BombyxmoriLinnaeus*, Dry body caused by Beauveria bassiana、*Curcuma aeruginosa* Roxb. Dry rhizome.
《马培之医案》	通络利温汤	*Glycine arenaria* Tindale, Dry processed products after seed germination、*Stephania tetrandra* S.Moore. Dry root、*Paeonia lactiflora* PalL, Dry root、*Gentiana macrophylla* Pall. Dry root、*Cyathula achyranthoides* (Kunth) Moq. Dry root、*Pheretima aspergillum* E.Perrier、*Angelica acutiloba* (Siebold & Zucc.) Kitag. Dry tail of the root、*Morus alba* L. Dry twig、*Phellodendron amurense* Rupr. Dried Root.
《景岳全书》卷五十四	愈风燥湿化痰丸	*Cyathula achyranthoides* (Kunth) Moq. Dry rhizome,fried、*Atractylodes lancea* （Thunb.）DC, Dry rhizome,Soaking rice water、*Eucommia ulmoides* Oliv, Fried ginger、*Achyranthes bidentata* Blume, Dried root、*Ligusticum acuminatum* Franch. Dry rhizome、*Coix lacryma-jobi* L. Dry mature seeds、*Psoralea corylifolia* L. Dry mature fruit、*Angelica sinensis* (Oliv.) Diels. Dried Root、*Gleditsia sinensis* Lam. Dry sterile fruit、*Saposhnikovia divaricata* （Turcz.） Schischk, Dried Root、*Notopterygium incisum* K.C.Ting ex H.T.Chang, Dry rhizome and root、*Angelica pubescens* Maxim. Dried root、*Rehmannia glutinosa* (Gaertn.) DC. Dry root、*Stephania tetrandra* S.Moore. Dry root、*Pinellia tripartita* (Blume) Schott, Dry blocky stem、*Arisaema agasthyanum* Sivad. & Sath.Kumar, Dry blocky stem、*Gastrodia elata* Bl. Dry blocky stem、*Aquilaria sinensis* （ Lour.）

		Gilg, Resin-containing wood、*Aucklandia lappa* DC. Dry root、*Citrus reticulata* Blanco, Dried fruit peel、*Aconitum abietetorum* W.T.Wang & L.Q.Li, Dried root、*BombyxmoriLinnaeus*, Dry body caused by Beauveria bassiana、*Buthus martensii* Karsch, Dry body.
《百一》卷三	大防风汤	*Saposhnikovia divaricata* （Turcz.） Schischk, Dried Root、*Cyathula achyranthoides* (Kunth) Moq. Dry rhizome、*Eucommia ulmoides Oliv. Dry bark*、Angelica sinensis *(Oliv.) Diels. Dried Root*、*Rehmannia glutinosa* (Gaertn.) DC. Dry root，*Steamed*、*Paeonia lactiflora* Pall. Dried root、*Astragalus aaronii* (Eig) Zohary, Dried Root、*Notopterygium incisum* K.C.Ting ex H.T.Chang, Dry rhizome and root、*Achyranthes bidentata* Blume, Dried root、*Glycyrrhiza uralensis* Fisch. Dried Root、*Panax ginseng* C.A.Mey. Dried Root、*Ligusticum acuminatum* Franch. Dry rhizome.
《宋•太平惠民和剂局方》	大防风汤	*Saposhnikovia divaricata* （Turcz.） Schischk, Dried Root、*Cyathula achyranthoides* (Kunth) Moq. Dry rhizome、*Eucommia ulmoides Oliv. Dry bark*、Angelica sinensis *(Oliv.) Diels. Dried Root*、*Rehmannia glutinosa* (Gaertn.) DC. Dry root，*Steamed*、*Paeonia lactiflora* Pall. Dried root、*Astragalus aaronii* (Eig) Zohary, Dried Root、*Notopterygium incisum* K.C.Ting ex H.T.Chang, Dry rhizome and root、*Achyranthes bidentata* Blume, Dried root、*Glycyrrhiza uralensis* Fisch. Dried Root、*Panax ginseng* C.A.Mey. Dried Root、*Ligusticum acuminatum* Franch. Dry rhizome.

	陈国藩方	咳喘止血汤	*Rehmannia glutinosa* (Gaertn.) DC. Dry root、*Agrimonia pilosa* Ledeb. Dry aerial part、*Stemona sessilifolia*（Miq.）Miq. Dry root、*Bletilla striata* (Thunb.) Rchb.f., Dry root、*Lilium brownii* F.E.Brown var.viridulum Baker, Dry fleshy linden、*Asparagus cochinchinensis*（Lour.）Merr.、*Glehnia littoralis* F.Schmidt ex Miq. Dry root、*Panax notoginseng* (Burkill) F.H.Chen, Dry rhizome and root, powder.
	《中医妇科治疗学》	冬地百部饮	*Rehmannia glutinosa* (Gaertn.) DC. Dry root、*Stemona japonica* (Blume) Miq. Dry root、*Asparagus cochinchinensis*（Lour.） Merr.、*Eriobotrya japonica*（Thunb.） Lindl. Dry leaf、*Fritillaria thunbergii* Miq. Dry bulb、*Ligustrum lucidum* W.T.Aiton, Dry mature fruit、*Imperata cylindrica* (L.) Raeusch. Dry rhizome.
Nourishing Yin and invigorating Yang	《中国医学大辞典》	萃仙丸	*Polygonum multiflorum* Thunb. Dry root、*Lycium barbarum* L. Dry mature fruit、*Euryale ferox* Salisb. Dry mature kernels、*Nelumbo nucifera* Gaertn. Dried stamen、*Dioscotea opposita* Thunb.、*Astragalus complanatus* Bunge, Dry mature seed、*Tribulus terrestris* L. Dry mature fruit、*Cuscuta chinensis* Lam. Dry mature seed、*Psoralea corylifolia* L. Dry mature fruit、*Allium aaseae* Ownbey, Dry mature seed、*Rubus chingii* Hu, Dry fruit、*Ginkgo biloba* L. Dry flesh、*Dipsacus asper* Wall. ex C.B. Clarke, Dry root、*Panax ginseng* C.A.Mey. Dried Root.
	《中国药典》	心舒宁片	*Ginkgo biloba* L. Dry leave、*Pueraria alopecuroides* Craib, Dry root、*Siegesbeckiaorientalis* L.Dry aerial part、*Diospyros kaki* L.f., Dry leaf.
	孟百三方	宁心汤	*Panax ginseng* C.A.Mey. Dried Root、*Salvia miltiorrhiza* Bunge, Dry rhizome and root、*Angelica sinensis* (Oliv.) Diels. Dried Root、*Ligusticum acuminatum* Franch. Dry rhizome、*Rehmannia glutinosa* (Gaertn.) DC. Dry root、*Paeonia lactiflora* PalL, Dry root、*Paeonia lactiflora* Pall. Dried root、*Prunus persica* (L.) Batsch, Dry kernels、*Carthamus tinctorius* L. Dry flower、*Poria cocos*（Schw.）Wolf, Dried

		Root、*Aucklandia lappa* DC. Dry root、*Citrus reticulata* Blanco, Dried fruit peel、*Glycyrrhiza uralensis* Fisch. Dried Root, Honeyed.
杨百方	凝冰散	*Glycyrrhiza uralensis* Fisch. Dried Root, Honeyed、*Astragalus aaronii* (Eig) Zohary, Dried Root、*Codonopsis pilosula*（Franch.）Nannf, Dry root、*Rehmannia glutinosa* (Gaertn.) DC. Dry root、*Ophiopogon japonicus* (L.f) Ker-GawL、*Poria cocos*（Schw.）Wolf, Dried Root、*Cinnamomum cassia* (L.) J.Presl, Dry twig、*Paeonia lactiflora* Pall. Dried root、*Curcuma longa* L, Dry root、*Citrus medica* L. Dried fruit、*Zingiber officinale* Roscoe, Fresh rhizome、*Ziziphus jujub*a Mill. Dry mature fruit.
《医醇剩义》卷二	苍玉潜龙汤	*Rehmannia glutinosa* (Gaertn.) DC. Dry root、*Chinemys reevesii* (Gray), Belly armor、*Chinemys reevesii* (Gray), Belly armor、*Moutan officinalis* (L.) Lindl. & Paxton, cortex、Saiga tatarica Linnaeus、*Glehnia littoralis* F.Schmidt ex Miq. Dry root、*Paeonia lactiflora* Pall. Dried root
《医学人门》卷七	杜仲丸	*Eucommia ulmoides* Oliv. Dry bark、*Chinemys reevesii* (Gray), Belly armor、*Phellodendron amurense* Rupr. Dried Root、*Dioscotea opposita* Thunb.、*Lycium barbarum* L. Dry mature fruit、*Schisandra arisanensis* Hayata, Dry ripe fruit、*Angelica sinensis* (Oliv.) Diels. Dried Root、*Paeonia lactiflora* Pall, Dry root、*Astragalus aaronii* (Eig) Zohary, Dried Root、*Psoralea corylifolia* L. Dry mature fruit.
