# Supplementary material for: System Prediction and Validation of TCM for Chronic Myeloid Leukemia Treatment from the Perspective of Low-Toxicity Chemotherapy: A Stilbene α-Viniferin Has a Proapoptotic Effect on K562 Cells via the Mitochondrial Pathway
Source: Evid Based Complement Alternat Med. 2020 Feb 10;2020:1986962. doi: 10.1155/2020/1986962 (PMC7035520; doi:10.1155/2020/1986962)
Supplement: Supplementary Materials — Supplemental Table 1: TCM prescriptions for CML. Supplemental Table 2: full ADME properties in silico of 121 candidate compounds via admetSAR Prediction. Supplemental Table 3: intersection of CML targets and prediction Targets. Supplemental Table 4: literature mining of 32 active compounds. Supplemental Table 5: NCI Cancer Screen Data of 32 candidate compounds on K562 cells. Supplemental Figure 1: effects of α-viniferin on peripheral blood mononuclear cells (PBMC) proliferation. Supplemental Figure 2: evaluation of α-viniferin on the protein expression level of BCR-ABL. Supplemental Figure 3: evaluation of α-viniferin on the mRNA level of caspase-9. [file 1986962.f1.zip › Supplementary Materials/Supplemental Table-2.pdf]

Supplemental Table-2. Full ADME properties in silico of 121 candidate compounds via admetSAR Prediction

Compounds	Absorption		Metabolism (CYP450 isoforms Inhibitor)						
	HIA	Caco-2	1A2	2C9	2D6	2C19	3A4	score	Herbs
Majudin	0.9933	0.6835	0.9774	0.8257	0.8931	0.9296	0.7959	3.48407919	Notopterygium incisum K.C.Ting ex H.T.Chang
Bergaptin	0.9964	0.5379	0.9107	0.8949	0.8931	0.8994	0.8123	3.47147818	Notopterygium incisum K.C.Ting ex H.T.Chang
sesamin	1	0.5774	0.9106	0.8949	0.8932	0.8994	0.796	3.46096872	Stemona sessilifolia (Miq.) Miq.
suchilactone	0.9884	0.7833	0.6598	0.9399	0.8096	0.9801	0.9591	3.39143188	Stemona sessilifolia (Miq.) Miq.
isorhamnetin	0.9783	0.8866	0.9218	0.756	0.6993	0.8648	0.7348	3.12557861	Ginkgobiloba.
Ammidin	0.9962	0.5187	0.8503	0.7199	0.7115	0.8977	0.7497	3.08492296	Notopterygium incisum K.C.Ting ex H.T.Chang
8-geranoxy-5-methoxypsoralen	0.9924	0.5556	0.8058	0.7084	0.788	0.8398	0.7848	3.0808701	Notopterygium incisum K.C.Ting ex H.T.Chang
Cnidilin	0.9925	0.5243	0.8169	0.5477	0.641	0.8984	0.6951	2.82470425	Notopterygium incisum K.C.Ting ex H.T.Chang
naringenin	0.967	0.7533	0.9106	0.8949	-0.7463	0.8994	0.8988	2.12533827	Smilax glabra Roxb.
Genkwanin	0.9921	0.929	0.9378	0.9385	-0.8253	0.9012	0.7232	2.00309141	Ginkgobiloba.
Diosmetin	0.9783	0.8866	0.9218	0.756	-0.6993	0.8648	0.7348	1.92963575	Ginkgobiloba.
Chryseriol	0.9783	0.8866	0.9218	0.756	-0.6993	0.8648	0.7348	1.92963575	Ginkgobiloba.
Kaempferid	0.9783	0.8866	0.9218	0.756	-0.6993	0.8648	0.7348	1.92963575	Smilax china L.
Syringetin	0.9719	0.8896	0.8668	0.6258	-0.6249	0.8187	0.7817	1.83717389	Ginkgobiloba.
4'-methylpinosylvin	0.9968	0.9191	0.9614	0.903	-0.8899	0.886	0.5	1.78240889	Stemona sessilifolia (Miq.) Miq.
Dehydrodieug	0.9721	0.8004	0.744	0.797	-0.7521	0.9261	0.6365	1.75847413	Eucommia

enol									ulmoides Oliv.
Moracin M	0.9964	0.6043	0.9061	0.7842	-0.8215	0.7031	0.804	1.74936332	Smilax china L.
kaempferol	0.9855	0.7447	0.9108	0.8948	-0.9083	0.6434	0.7241	1.66803093	Eucommia ulmoides Oliv.; Moutan officinalis (L.) Lindl. & Paxton, cortex; Morus alba L., twig, dry; Ginkgobiloba.
morin	0.9855	0.7447	0.9108	0.8948	-0.9083	0.6434	0.7241	1.66803093	Morus alba L., twig, dry
notoptol	0.9762	0.5537	0.7233	0.7758	-0.6654	0.7588	0.6368	1.66419893	Notopterygium incisum K.C.Ting ex H.T.Chang
Dihydroresveratrol	0.9503	0.8604	0.7749	0.742	-0.8326	0.8434	0.7201	1.65802923	Smilax glabra Roxb.
cis-resveratrol	0.9952	0.8915	0.9106	0.7068	-0.9226	0.8052	0.7539	1.65443638	Smilax china L.; Reynoutria japonica Houtt.
cis-pinosylvin	0.9948	0.9014	0.9177	0.6648	-0.9171	0.8322	0.6678	1.5974595	Eucommia ulmoides Oliv.
Oxysanguinarine	0.9841	0.7216	0.8373	-0.8557	0.5126	0.7982	0.669	1.50874759	Morus alba L., twig, dry
α-Viniferin	1	0.6216	0.9277	0.9497	-0.7239	0.8042	-0.6358	1.13587144	Paeonia anomala L.; Moutan officinalis (L.) Lindl. & Paxton, cortex; Reynoutria japonica Houtt.; Ginkgobiloba.
AIDS214634	0.5176	0.6408	0.6819	0.5427	-0.8266	0.5876	0.542	1.10669817	Eucommia ulmoides Oliv.
Isoflavon	1	0.8727	0.9662	0.7441	-0.9436	0.9213	-0.6675	0.88838768	Ginkgobiloba.
4,7-Dihydroxy-5-methoxyl-6-methyl-8-formyl-flavan	0.9889	0.9077	0.7694	0.565	-0.6802	0.7659	-0.6223	0.71368052	Smilax glabra Roxb.
6,8-Dihydroxy-7-methoxyxanthone	0.9306	0.9044	0.9724	-0.6549	-0.6476	0.6923	0.6321	0.67863562	Reynoutria japonica Houtt.

40957-99-1	0.9944	0.763	-0.5381	0.6912	-0.8313	0.7443	0.5296	0.34602068	Eucommia ulmoides Oliv.
(+)-medioresinol	0.9944	0.763	-0.5381	0.6912	-0.8313	0.7443	0.5296	0.34602068	Eucommia ulmoides Oliv.
Torachrysone	0.9803	0.92	0.9469	-0.7326	-0.8119	0.5056	0.5459	0.2491008	Reynoutria japonica Houtt.
palmidin A	0.9932	0.6209	0.7561	0.7273	-0.7856	-0.5209	-0.5122	-0.22252461	Rheum palmatum L.
aloe-emodin	0.9815	0.5847	0.7957	0.6696	-0.766	-0.5	-0.7395	-0.34954203	Rheum palmatum L.
Morkit	1	0.8682	0.9357	0.5919	-0.6483	-0.6245	-0.9241	-0.41647792	Reynoutria japonica Houtt.
EUPATIN	0.9854	0.8992	0.8642	-0.6863	-0.8329	0.6566	-0.7237	-0.49638525	Rheum palmatum L.
Xanthotoxol	0.9811	0.6754	0.8392	-0.7372	-0.5847	-0.6555	0.627	-0.53089239	Notopterygium incisum K.C.Ting ex H.T.Chang
Pterostilbene	0.9974	0.8911	0.8736	-0.9304	-0.9249	0.7583	-0.5979	-0.60006547	Notopterygium incisum K.C.Ting ex H.T.Chang
Toralactone	0.808	0.9237	0.7076	-0.6892	-0.7022	-0.7325	0.6957	-0.71779906	Rheum palmatum L.
Laricitrin	0.9482	0.6824	0.9091	-0.7982	-0.8687	-0.6361	0.633	-0.7462083	Ginkgobiloba.
didehydrotuberostemonine	1	0.6682	0.7281	-0.5909	-0.8438	-0.6156	0.5101	-0.76892367	Stemona sessilifolia (Miq.) Miq.
luteolin	0.965	0.8957	0.9106	-0.5823	-0.9287	-0.9025	0.6951	-0.79768769	Reynoutria japonica Houtt.; Ginkgobiloba.
quercetin	0.965	0.8957	0.9106	-0.5823	-0.9287	-0.9025	0.6951	-0.79768769	Eucommia ulmoides Oliv.; Reynoutria japonica Houtt.; Moutan officinalis (L.) Lindl. & Paxton, cortex; Smilax glabra Roxb.; Ginkgobiloba
(-)-taxifolin	0.965	0.8957	0.9106	-0.5823	-0.9287	-0.9025	0.6951	-0.79768769	Smilax glabra Roxb.
taxifolin	0.965	0.8957	0.9106	-0.5823	-0.9287	-0.9025	0.6951	-0.79768769	Smilax china L.;

cis-Dihydroquercetin	0.965	0.8957	0.9106	-0.5823	-0.9287	-0.9025	0.6951	-0.79768769	Smilax glabra Roxb. Smilax china L.; Smilax glabra Roxb.
2-(4-hydroxyphenyl)ethyl 4-methoxybenzoate	0.913	0.8278	0.6793	-0.647	-0.9494	0.6062	-0.8666	-0.84789435	Notopterygium incisum K.C.Ting ex H.T.Chang
Procyanidin B-5,3'-O-gallate	0.6772	0.8786	-0.9306	-0.6236	-0.9414	0.5937	0.8413	-1.04234896	Rheum palmatum L.
Erythraline	0.9903	0.7737	-0.6103	-0.9082	0.6973	-0.7351	-0.6481	-1.63921898	Eucommia ulmoides Oliv.
bisdehydroneotuberostemonine	1	0.7947	0.8351	-0.7972	-0.8819	-0.7283	-0.5961	-1.684009	Stemona sessilifolia (Miq.) Miq.
Demethylfuropinnarin	0.986	0.5274	-0.757	-0.5341	-0.7866	-0.6967	0.8551	-1.72717362	Notopterygium incisum K.C.Ting ex H.T.Chang
(2R,8aS)-2,5,5,8a-tetramethyl-3,6-dihydro-2H-chromene	1	0.7072	-0.6951	-0.541	-0.896	0.6473	-0.782	-1.74929595	Ginkgobiloba.
bisdehydrostemoninine A	1	0.6195	0.5846	-0.6855	-0.8956	-0.6526	-0.693	-1.81177688	Stemona sessilifolia (Miq.) Miq.
bisdehydrostemoninine B	1	0.6195	0.5846	-0.6855	-0.8956	-0.6526	-0.693	-1.81177688	Stemona sessilifolia (Miq.) Miq.
Dipropyl phthalate	0.976	0.7171	0.6006	-0.6511	-0.8898	-0.5322	-0.9319	-1.82332052	Paeonia anomala L.
Picralinal	0.9523	0.5762	-0.6662	0.5	-0.8749	-0.5633	-0.7786	-1.84585895	Reynoutria japonica Houtt.
Guaiol	0.9971	0.6777	-0.6113	0.5515	-0.9198	-0.5347	-0.9156	-1.86356377	Notopterygium incisum K.C.Ting ex H.T.Chang
(Z)-1,3-di(phenyl)prop-2-en-1-one	0.9868	0.7267	-0.6793	0.7477	-0.9424	-0.6922	-0.8629	-1.87383447	Ginkgobiloba.
Mutatochrome	1	0.6964	-0.5529	-0.7813	-0.9208	0.5353	-0.8346	-1.97145643	Rheum palmatum L.

Moutan officinalis (L.) Lindl. & Paxton, cortexoside-h _qt 2	1	0.8465	0.7765	-0.9292	-0.9619	-0.5912	-0.9796	-2.04293318	Moutan officinalis (L.) Lindl. & Paxton, cortex
cis-Verbenyl acetate	1	0.7257	-0.8198	-0.8266	-0.8985	0.624	-0.8518	-2.14580769	Notopterygium incisum K.C.Ting ex H.T.Chang
rhein	0.9689	0.6092	-0.6497	0.5	-0.868	-0.9233	-0.9258	-2.21140421	Rheum palmatum L.; Reynoutria japonica Houtt.
stemonine	0.9969	0.6647	0.561	-0.8532	-0.9112	-0.8372	-0.832	-2.21713706	Stemona sessilifolia (Miq.) Miq.
stemoninine B	0.9969	0.6647	0.561	-0.8532	-0.9112	-0.8372	-0.832	-2.21713706	Stemona sessilifolia (Miq.) Miq.
Enhydrin	0.9483	0.5168	-0.5328	-0.8902	-0.9273	-0.8657	0.6247	-2.23507203	Smilax glabra Roxb.
dihydrostemoninine	0.7795	0.7545	0.7434	-0.9321	-0.9603	-0.8954	-0.9422	-2.29173649	Stemona sessilifolia (Miq.) Miq.
Physovenine	0.9963	0.5936	-0.6072	-0.7115	-0.7206	-0.6225	-0.61	-2.5761631	Reynoutria japonica Houtt.
Isogoycyrol	0.9421	0.5174	-0.6362	-0.6707	-0.7755	-0.7514	-0.7693	-2.82058551	Ginkgobiloba.
2H-3,9a-Methano-1-benzoxepin-9-methanol, octahydro-2,2,5a-trimethyl-, (3R-(3alpha,5aalpha,9alpha,9aalpha))	1	0.596	-0.7736	-0.5085	-0.9158	-0.5554	-0.9291	-2.86765646	Smilax glabra Roxb.
bis[(2S)-2-ethylhexyl] benzene-1,2-dicarboxylate	0.9797	0.7003	-0.6539	-0.7448	-0.8432	-0.631	-0.8309	-2.89507119	Ginkgobiloba.
(1R,2R)-1,2-Diphenylethyle	0.935	0.6935	-0.7233	-0.5862	-0.9209	-0.6888	-0.7833	-2.90673728	Ginkgobiloba.

nediamine									
olivil	0.9607	0.5748	-0.746	-0.7071	-0.9041	-0.6443	-0.7101	-2.92474861	Eucommia ulmoides Oliv.
6'-Feruloylnodakenin	0.593	0.8264	-0.7356	-0.6762	-0.8667	-0.5911	-0.9088	-2.94483359	Notopterygium incisum K.C.Ting ex H.T.Chang
DBP	0.9941	0.6564	-0.592	-0.897	-0.5245	-0.9265	-0.8539	-2.94698555	Ginkgobiloba.
PIT	0.7874	0.5717	-0.6416	-0.7426	-0.8602	-0.7117	-0.8345	-2.9651409	Rheum palmatum L.
Marmesin	0.9881	0.7636	-0.6717	-0.7486	-0.827	-0.7313	-0.8285	-2.97800069	Notopterygium incisum K.C.Ting ex H.T.Chang
(8S)-8-(2-hydroxypropan-2-yl)-8,9-dihydrofuro[2,3-h]chromen-2-one	0.9881	0.7636	-0.6717	-0.7486	-0.827	-0.7313	-0.8285	-2.97800069	Notopterygium incisum K.C.Ting ex H.T.Chang
nodakenetin	0.9881	0.7636	-0.6717	-0.7486	-0.827	-0.7313	-0.8285	-2.97800069	Notopterygium incisum K.C.Ting ex H.T.Chang
copaene	1	0.6705	-0.8244	-0.6794	-0.9197	-0.5547	-0.8317	-2.98601895	Notopterygium incisum K.C.Ting ex H.T.Chang
DIBP	0.9745	0.7151	-0.5666	-0.6604	-0.9218	-0.7906	-0.8983	-2.99550166	Ginkgobiloba.
isoengelitin	0.9051	0.7493	-0.5306	-0.8538	-0.9547	-0.8339	-0.7109	-3.06337419	Smilax china L.; Smilax glabra Roxb.
Engelitin	0.9051	0.7493	-0.5306	-0.8538	-0.9547	-0.8339	-0.7109	-3.06337419	Smilax china L.
astilbin	0.9051	0.7493	-0.5306	-0.8538	-0.9547	-0.8339	-0.7109	-3.06337419	Smilax china L.; Smilax glabra Roxb.
Neoastilbin	0.9051	0.7493	-0.5306	-0.8538	-0.9547	-0.8339	-0.7109	-3.06337419	Smilax glabra Roxb.
β-cedrene	0.9954	0.6484	-0.7613	-0.7311	-0.9271	-0.7087	-0.8805	-3.13789978	Notopterygium incisum K.C.Ting ex H.T.Chang
diversoside_qt	0.6685	0.8787	-0.687	-0.7973	-0.9165	-0.7401	-0.918	-3.17110414	Notopterygium

			3						incisum K.C.Ting ex H.T.Chang
hirsutin_qt	0.8588	0.5	-0.6948	-0.8465	-0.8465	-0.7712	-0.964	-3.21152389	Eucommia ulmoides Oliv.
Daucosterol_qt	0.8788	0.8336	-0.8052	-0.7682	-0.9187	-0.8388	-0.7938	-3.24509009	Rheum palmatum L.
(-)-alpha-cedrene	1	0.6555	-0.7976	-0.7774	-0.929	-0.7584	-0.8897	-3.2521838	Paeonia anomala L.
nodakenin	0.7967	0.8705	-0.6932	-0.868	-0.9033	-0.8343	-0.8892	-3.27860349	Notopterygium incisum K.C.Ting ex H.T.Chang
benzoylpaeoniflorin	0.6197	0.8556	-0.8732	-0.8869	-0.9338	-0.7759	-0.8118	-3.3695257	Paeonia anomala L.
Torachrysone-8-O-beta-D-(6'-oxayl)-glucoside	0.7886	0.7989	-0.6882	-0.8912	-0.917	-0.9199	-0.908	-3.38591486	Rheum palmatum L.; Reynoutria japonica Houtt.
albiflorin_qt	0.7098	0.8668	-0.8927	-0.8984	-0.9399	-0.8197	-0.8046	-3.43048168	Paeonia anomala L.
paeoniflorin	0.8238	0.8487	-0.8834	-0.9006	-0.9314	-0.7995	-0.8545	-3.433317	Paeonia anomala L.
paeoniflorin_qt	0.8238	0.8487	-0.8834	-0.9006	-0.9314	-0.7995	-0.8545	-3.433317	Paeonia anomala L.; Moutan officinalis (L.) Lindl. & Paxton, cortex
4-O-methylpaeoniflorin_qt	0.6133	0.8067	-0.8816	-0.8915	-0.9323	-0.8	-0.8696	-3.43546595	Moutan officinalis (L.) Lindl. & Paxton, cortex
(3S,5R,8R,9R,10S,14S)-3,17-dihydroxy-4,4,8,10,14-pentamethyl-2,3,5,6,7,9-hexahydro-1H-cyclopenta[a]phenanthrene-15,16-dione	0.997	0.6592	-0.8825	-0.8505	-0.9601	-0.8399	-0.8531	-3.44859012	Paeonia anomala L.
paeonidanin_qt	0.6484	0.8203	-0.8945	-0.88	-0.9411	-0.8085	-0.868	-3.45009366	Moutan officinalis (L.)

									Lindl. & Paxton, cortex
paeoniflorgenone	0.9811	0.6976	-0.8872	-0.8919	-0.9494	-0.8415	-0.8208	-3.4569193	Paeonia anomala L.
diosgenin	0.9961	0.5872	-0.8638	-0.8878	-0.9107	-0.9127	-0.8309	-3.46533455	Smilax china L.; Smilax glabra Roxb.
Lactiflorin	0.5395	0.8577	-0.8997	-0.8963	-0.9457	-0.7886	-0.9046	-3.47891244	Paeonia anomala L.
L-Bornyl acetate	0.9969	0.7743	-0.9317	-0.9218	-0.9606	-0.7031	-0.9384	-3.49110903	Notopterygium incisum K.C.Ting ex H.T.Chang; Smilax glabra Roxb.
gallic acid-3-O-(6'-O-galloyl)-glucoside	0.8558	0.898	-0.9315	-0.7955	-0.9425	-0.9121	-0.8723	-3.49989554	Rheum palmatum L.
(-)-Tabernemontanine	1	0.5706	-0.9045	-0.907	-0.9231	-0.9025	-0.8309	-3.51627556	Eucommia ulmoides Oliv.
ent-Epicatechin	0.9654	0.8956	-0.9046	-0.9071	-0.9231	-0.9041	-0.8309	-3.51772585	Eucommia ulmoides Oliv.
(-)-catechin	0.9654	0.8956	-0.9046	-0.9071	-0.9231	-0.9041	-0.8309	-3.51772585	Rheum palmatum L.; Ginkgobiloba
(+)-catechin	0.9654	0.8956	-0.9046	-0.9071	-0.9231	-0.9041	-0.8309	-3.51772585	Paeonia anomala L.; Moutan officinalis (L.) Lindl. & Paxton, cortex; Reynoutria japonica Houtt.; Ginkgobiloba.
beta-sitosterol	1	0.7953	-0.9291	-0.9125	-0.9346	-0.9025	-0.8309	-3.55056073	Smilax china L.; Paeonia anomala L.; Rheum palmatum L.; Reynoutria japonica Houtt.; Smilax glabra Roxb.
sitosterol	1	0.7953	-0.9291	-0.9125	-0.9346	-0.9025	-0.8309	-3.55056073	Smilax china L.; Paeonia anomala

									L.; Moutan officinalis (L.) Lindl. & Paxton, cortex; Notopterygium incisum K.C.Ting ex H.T.Chang; Smilax glabra Roxb.
Stigmasterol	1	0.7953	-0.9291	-0.9125	-0.9346	-0.9025	-0.8309	-3.55056073	Smilax glabra Roxb.; Ginkgobiloba.
Mairin	0.9952	0.8835	-0.9167	-0.9071	-0.9554	-0.9025	-0.8309	-3.55391481	Paeonia anomala L.; Moutan officinalis (L.) Lindl. & Paxton, cortex
oleanolic acid	1	0.8353	-0.9169	-0.9071	-0.9485	-0.9025	-0.8695	-3.57307456	Smilax china L.; Paeonia anomala L.; Moutan officinalis (L.) Lindl. & Paxton, cortex
pseudoprotodioscin_qt	0.6254	0.8883	-0.8848	-0.9118	-0.936	-0.9113	-0.946	-3.59643242	Smilax china L.
methylprotodioscin_qt	0.5247	0.8488	-0.8921	-0.9148	-0.9364	-0.8976	-0.9573	-3.60138169	Smilax china L.
1-O-Galloyl-glycerol	0.7501	0.8336	-0.7642	-0.973	-0.9622	-0.9726	-0.9526	-3.6232814	Rheum palmatum L.
Physciondiglucoside	0.7916	0.8674	-0.9167	-0.9167	-0.9519	-0.9109	-0.9311	-3.6300136	Rheum palmatum L.; Reynoutria japonica Houtt.
Luteolin-4′-glucoside	0.7855	0.9398	-0.9084	-0.9296	-0.9513	-0.9289	-0.9193	-3.63996795	Ginkgobiloba.
Cudranin	0.7855	0.9398	-0.9084	-0.9296	-0.9513	-0.9289	-0.9193	-3.63996795	Smilax china L.; Morus alba L., twig, dry
Sennoside E_qt	0.5493	0.9338	-0.932	-0.9171	-0.9596	-0.9032	-0.9365	-3.64666492	Rheum palmatum L.
Sennoside D_qt	0.5493	0.9338	-0.932	-0.9171	-0.9596	-0.9032	-0.9365	-3.64666492	Rheum palmatum L.
6-Hydroxykyn	0.9193	0.6778	-0.897	-0.9167	-0.9492	-0.9641	-0.9666	-3.67756296	Ginkgobiloba.

urenate	2
