# Supplementary material for: System Prediction and Validation of TCM for Chronic Myeloid Leukemia Treatment from the Perspective of Low-Toxicity Chemotherapy: A Stilbene α-Viniferin Has a Proapoptotic Effect on K562 Cells via the Mitochondrial Pathway
Source: Evid Based Complement Alternat Med. 2020 Feb 10;2020:1986962. doi: 10.1155/2020/1986962 (PMC7035520; doi:10.1155/2020/1986962)
Supplement: Supplementary Materials — Supplemental Table 1: TCM prescriptions for CML. Supplemental Table 2: full ADME properties in silico of 121 candidate compounds via admetSAR Prediction. Supplemental Table 3: intersection of CML targets and prediction Targets. Supplemental Table 4: literature mining of 32 active compounds. Supplemental Table 5: NCI Cancer Screen Data of 32 candidate compounds on K562 cells. Supplemental Figure 1: effects of α-viniferin on peripheral blood mononuclear cells (PBMC) proliferation. Supplemental Figure 2: evaluation of α-viniferin on the protein expression level of BCR-ABL. Supplemental Figure 3: evaluation of α-viniferin on the mRNA level of caspase-9. [file 1986962.f1.zip › Supplementary Materials/Supplemental Table-3.pdf]

Supplemental Table-3. Intersection of CML Targets and Prediction Targets

OFFICIAL_GENE_SYMBOL	Gene Name	Related Genes	Species
ABCB1	ATP binding cassette subfamily B member 1(ABCB1)	RG	Homo sapiens
ACHE	acetylcholinesterase (Cartwright blood group)(ACHE)	RG	Homo sapiens
ADRB1	adrenoceptor beta 1(ADRB1)	RG	Homo sapiens
ADRB2	adrenoceptor beta 2(ADRB2)	RG	Homo sapiens
ALOX5	arachidonate 5-lipoxygenase(ALOX5)	RG	Homo sapiens
AR	androgen receptor(AR)	RG	Homo sapiens
BCL2	BCL2, apoptosis regulator(BCL2)	RG	Homo sapiens
CA2	carbonic anhydrase 2(CA2)	RG	Homo sapiens
CCL2	C-C motif chemokine ligand 2(CCL2)	RG	Homo sapiens
DRD3	dopamine receptor D3(DRD3)	RG	Homo sapiens
ECE1	endothelin converting enzyme 1(ECE1)	RG	Homo sapiens
EGF	epidermal growth factor(EGF)	RG	Homo sapiens
EGFR	epidermal growth factor receptor(EGFR)	RG	Homo sapiens
F2	coagulation factor II, thrombin(F2)	RG	Homo sapiens
F7	coagulation factor VII(F7)	RG	Homo sapiens
G6PD	glucose-6-phosphate dehydrogenase(G6PD)	RG	Homo sapiens
GABRA2	gamma-aminobutyric acid type A receptor alpha2 subunit(GABRA2)	RG	Homo sapiens
GSTM1	glutathione S-transferase mu 1(GSTM1)	RG	Homo sapiens
HGF	hepatocyte growth factor(HGF)	RG	Homo sapiens
HTR2A	5-hydroxytryptamine receptor 2A(HTR2A)	RG	Homo sapiens
IFNG	interferon gamma(IFNG)	RG	Homo sapiens
IL1B	interleukin 1 beta(IL1B)	RG	Homo sapiens
IL6	interleukin 6(IL6)	RG	Homo sapiens
INSR	insulin receptor(INSR)	RG	Homo sapiens
KCNH2	potassium voltage-gated channel subfamily H member 2(KCNH2)	RG	Homo sapiens
KDR	kinase insert domain receptor(KDR)	RG	Homo sapiens
LDLR	low density lipoprotein receptor(LDLR)	RG	Homo sapiens
MMP1	matrix metallopeptidase 1(MMP1)	RG	Homo sapiens
MMP2	matrix metallopeptidase 2(MMP2)	RG	Homo sapiens
MMP3	matrix metallopeptidase 3(MMP3)	RG	Homo sapiens
MPO	myeloperoxidase(MPO)	RG	Homo sapiens
MTOR	mechanistic target of rapamycin(MTOR)	RG	Homo sapiens
NOS3	nitric oxide synthase 3(NOS3)	RG	Homo sapiens
NQO1	NAD(P)H quinone dehydrogenase 1(NQO1)	RG	Homo sapiens
NQO2	NAD(P)H quinone dehydrogenase 2(NQO2)	RG	Homo sapiens
NR3C1	nuclear receptor subfamily 3 group C member 1(NR3C1)	RG	Homo sapiens
PLAU	plasminogen activator, urokinase(PLAU)	RG	Homo sapiens
PPARG	peroxisome proliferator activated receptor gamma(PPARG)	RG	Homo sapiens

PTPN1	protein tyrosine phosphatase, non-receptor type 1(PTPN1)	RG	Homo sapiens
PYGM	phosphorylase, glycogen, muscle(PYGM)	RG	Homo sapiens
SCN5A	sodium voltage-gated channel alpha subunit 5(SCN5A)	RG	Homo sapiens
SELE	selectin E(SELE)	RG	Homo sapiens
SLC6A3	solute carrier family 6 member 3(SLC6A3)	RG	Homo sapiens
SLC6A4	solute carrier family 6 member 4(SLC6A4)	RG	Homo sapiens
SOD1	superoxide dismutase 1, soluble(SOD1)	RG	Homo sapiens
THBD	thrombomodulin(THBD)	RG	Homo sapiens
TNF	tumor necrosis factor(TNF)	RG	Homo sapiens
TOP1	topoisomerase (DNA) I(TOP1)	RG	Homo sapiens
TP53	tumor protein p53(TP53)	RG	Homo sapiens
