# Supplementary material for: System Prediction and Validation of TCM for Chronic Myeloid Leukemia Treatment from the Perspective of Low-Toxicity Chemotherapy: A Stilbene α-Viniferin Has a Proapoptotic Effect on K562 Cells via the Mitochondrial Pathway
Source: Evid Based Complement Alternat Med. 2020 Feb 10;2020:1986962. doi: 10.1155/2020/1986962 (PMC7035520; doi:10.1155/2020/1986962)
Supplement: Supplementary Materials — Supplemental Table 1: TCM prescriptions for CML. Supplemental Table 2: full ADME properties in silico of 121 candidate compounds via admetSAR Prediction. Supplemental Table 3: intersection of CML targets and prediction Targets. Supplemental Table 4: literature mining of 32 active compounds. Supplemental Table 5: NCI Cancer Screen Data of 32 candidate compounds on K562 cells. Supplemental Figure 1: effects of α-viniferin on peripheral blood mononuclear cells (PBMC) proliferation. Supplemental Figure 2: evaluation of α-viniferin on the protein expression level of BCR-ABL. Supplemental Figure 3: evaluation of α-viniferin on the mRNA level of caspase-9. [file 1986962.f1.zip › Supplementary Materials/Supplemental Table-4.pdf]

Supplemental Table-4. Literature mining of 32 candidate compounds

Classification	Compound	CID	Literatures
Flavones			
	naringenin	932	Li R , Feng Y , Chen J , et al. Naringenin suppresses K562 human leukemia cell proliferation and ameliorates Adriamycin-induced oxidative damage in polymorphonuclear leukocytes[J]. Experimental and Therapeutic Medicine, 2015, 9(3):697---706.<br>Liu X , Ye F , Wu J , et al. Signaling Proteins and Pathways Affected by Flavonoids in Leukemia Cells[J]. Nutrition and Cancer, 2015, 67(2):238-249.
	Isoflavon	72304	Zhang S J , Sun D , Hao J B , et al. The effect of dietary soyabean isoflavones on photodynamic therapy in K562 leukemia cells[J]. Journal of Photochemistry & Photobiology B Biology, 2012, 110(none):28-33.<br>Zhang D , Zhuang Y , Pan J , et al. Investigation of Effects and Mechanisms of Total Flavonoids of\r, *Astragalus*\r, and Calycosin on Human Erythroleukemia Cells[J]. Oxidative Medicine and Cellular Longevity, 2012, 2012:1-5.
	4,7-Dihydroxy-5-methoxyl-6-methyl-8-formyl-flavan	129394	Zhang D , Zhuang Y , Pan J , et al. Investigation of Effects and Mechanisms of Total Flavonoids of\r, *Astragalus*\r, and Calycosin on Human Erythroleukemia Cells[J]. Oxidative Medicine and Cellular Longevity, 2012, 2012:1-5.
	Chryseriol	5280666	Ninomiya M , Nishida K , Kaori Tanaka…. Structure–activity relationship studies of 5,7-dihydroxyflavones as naturally occurring inhibitors of cell proliferation in human leukemia HL-60 cells[J]. Journal of Natural Medicines, 2013, 67(3):460-467.<br>Gatouillat, Grégory, Alabdul Magid A , Bertin E , et al. Cytotoxicity and Apoptosis Induced by Alfalfa (\r, Medicago sativa\r,) Leaf Extracts in Sensitive and Multidrug-Resistant Tumor Cells[J]. Nutrition and Cancer, 2014, 66(3):483-491.
	kaempferol	5280863	Marfe G , Tafani M , Indelicato M , et al. Kaempferol induces apoptosis in Two different cell lines Via Akt inactivation, Bax and SIRT3 activation, and mitochondrial dysfunction[J]. Journal of Cellular Biochemistry, 2009, 106(4):643-650.<br>Okoye F B C , Sawadogo W R , Sendker J , et al. Flavonoid glycosides from Olax mannii: Structure elucidation and effect on the nuclear factor kappa B pathway[J]. Journal of Ethnopharmacology, 2015, 176(Complete):27-34.
	Diosmetin	5281612	Roma A , Rota S G , Spagnuolo P A . Diosmetin Induces Apoptosis of Acute Myeloid Leukemia Cells[J]. Molecular Pharmaceutics, 2018:acs.molpharmaceut.7b01151.
	Genkwanin	5281617	Sghaier M B , Skandrani I , Nasr N , et al. Flavonoids and sesquiterpenes from Tecurium ramosissimum promote antiproliferation of human cancer cells and enhance antioxidant activity: A structure–activity relationship study[J]. Environ Toxicol Pharmacol, 2011, 32(3):0-348.<br>Zahir A , Jossang A , Bodo B , et al. DNA topoisomerase I inhibitors:

			cytotoxic flavones from Lethedon tannaensis[J]. Journal of Natural Products, 1996, 59(7):701-3.
	Kaempferid	5281666	not founded
	morin	5281670	Park C , Lee W S , Go S I , et al. Morin, a Flavonoid from Moraceae, Induces Apoptosis by Induction of BAD Protein in Human Leukemic Cells[J]. International Journal of Molecular Sciences, 2014, 16(1):645.<br>Ikegawa T , Ohtani H , Koyabu N , et al. Inhibition of P-glycoprotein by flavonoid derivatives in adriamycin-resistant human myelogenous leukemia (K562/ADM) cells[J]. Cancer Letters, 2002, 177(1):89-93.
	Syringetin	5281953	Hibasami H , Mitani A , Katsuzaki H , et al. Isolation of five types of flavonol from seabuckthorn (Hippophae rhamnoides) and induction of apoptosis by some of the flavonols in human promyelotic leukemia HL-60 cells[J]. International Journal of Molecular Medicine, 2005, 15(5):805---809.
	6,8-Dihydroxy-7-methoxyxanthone	5316798	not founded
	isorhamnetin	5318645	Wu Xunxun,Chen Xiaofei,Dan Jia,Cao Yan,Gao Shouhong,Guo Zhiying,Zerbe Philipp,Chai Yifeng,Diao Yong,Zhang Lei. Characterization of anti-leukemia components from Indigo naturalis using comprehensive two-dimensional K562/cell membrane chromatography and in silico target identification.[J]. Scientific reports,2016,6.<br>Hibasami H , Mitani A , Katsuzaki H , et al. Isolation of five types of flavonol from seabuckthorn (Hippophae rhamnoides) and induction of apoptosis by some of the flavonols in human promyelotic leukemia HL-60 cells[J]. International Journal of Molecular Medicine, 2005, 15(5):805---809.
	suchilactone	132350840	not founded
stilbenes			
	Dihydroresveratrol	185914	not founded
	α-Viniferin	196402	not founded
	cis-resveratrol	1548910	Thikrayat A A , Madihally S V . Influence of controlled release of resveratrol from electrospun fibers in combination with siRNA on leukemia cells[J]. European Journal of Pharmaceutical Sciences, 2018, 123:173-183.<br>Wu X P , Xiong M , Xu C S , et al. Resveratrol induces apoptosis of human chronic myelogenous leukemia cells in vitro through p38 and JNK-regulated H2AX phosphorylation[J]. Acta Pharmacologica Sinica, 2015, 36(3):353-361.
	cis-pinosylvin	9548840	not founded
	4'-methylpinosylvin	44566996	not founded
Coumarins			
	Majudin	2355	not founded

	Ammidin	10212	not founded
	Cnidilin	821449	not founded
	notoptol	5320228	not founded
	Bergaptin	5471349	not founded
	8-geranoxy-5-methoxy psoralen	6442182	not founded
Others			
Biphenyls	Dehydrodieugenol	165225	not founded
Lignin	(+)-medioresinol （40957-99-1）	181681	Park H J , Lee M S , Lee K T , et al. Studies on Constituents with Cytotoxic Activity from the Stem Bark of Syringa velutina.[J]. CHEMICAL & PHARMACEUTICAL BULLETIN, 1999, 47(7):1029-1031.
benzofuran	Moracin M	185848	not founded
Quinones	AIDS214634	214634	not founded
Hydroxyhemo globin	Oxysanguinarine	443716	not founded
Solanine	Torachrysone	5321977	not founded
