# Supplementary material for: System Prediction and Validation of TCM for Chronic Myeloid Leukemia Treatment from the Perspective of Low-Toxicity Chemotherapy: A Stilbene α-Viniferin Has a Proapoptotic Effect on K562 Cells via the Mitochondrial Pathway
Source: Evid Based Complement Alternat Med. 2020 Feb 10;2020:1986962. doi: 10.1155/2020/1986962 (PMC7035520; doi:10.1155/2020/1986962)
Supplement: Supplementary Materials — Supplemental Table 1: TCM prescriptions for CML. Supplemental Table 2: full ADME properties in silico of 121 candidate compounds via admetSAR Prediction. Supplemental Table 3: intersection of CML targets and prediction Targets. Supplemental Table 4: literature mining of 32 active compounds. Supplemental Table 5: NCI Cancer Screen Data of 32 candidate compounds on K562 cells. Supplemental Figure 1: effects of α-viniferin on peripheral blood mononuclear cells (PBMC) proliferation. Supplemental Figure 2: evaluation of α-viniferin on the protein expression level of BCR-ABL. Supplemental Figure 3: evaluation of α-viniferin on the mRNA level of caspase-9. [file 1986962.f1.zip › Supplementary Materials/Supplemental Table-5.pdf]

Supplemental Table-5. NCI Cancer Scree date of 32 candidate compounds on K562

Compound	$LogGI_{50}$	One dose（10μM）<br>Growth percent%
Majudin	-4	
Ammidin		-
Cnidilin		-
notoptol		-
Bergaptin		-
8-geranoxy-5-methoxypsoralen		-
naringenin		97.6
Isoflavon	-4	
4,7-Dihydroxy-5-methoxyl-6-methyl-8-formyl-flavan		106.2
Chryseriol		-
kaempferol	-4	
Diosmetin	-4	
Genkwanin		-
Kaempferid		-
morin	-4	
Syringetin		-
6,8-Dihydroxy-7-methoxyxanthone		115.8
isorhamnetin		-
suchilactone		-
Dihydroresveratrol	-4.1	
α-Viniferin	-5.2	
cis-resveratrol		-
cis-pinosylvin		89
4'-methylpinosylvin		-
Dehydrodieugenol		-
(+)-medioresinol（40957-99-1）		-
Moracin M		-
AIDS214634		-
Oxysanguinarine		-
Torachrysone		-
